# Genome-wide screening for genetic variants in polyadenylation signal (PAS) sites in mouse selection lines for fatness and leanness

**DOI:** 10.1007/s00335-022-09967-8

**Published:** 2022-11-21

**Authors:** Martin Šimon, Špela Mikec, Nicholas M. Morton, Santosh S. Atanur, Janez Konc, Simon Horvat, Tanja Kunej

**Affiliations:** 1 Biotechnical Faculty, Department of Animal Science, University of Ljubljana, Domžale, Slovenia; 2 University of Edinburgh, The Queen’s Medical Research Institute, Centre for Cardiovascular Science, Edinburgh, UK; 3 Faculty of Medicine, Department of Metabolism, Digestion and Reproduction, Imperial College London, London, UK; 4 Centre for Genomic and Experimental Medicine, University of Edinburgh, Edinburgh, UK; 5 Laboratory for Molecular Modeling, National Institute of Chemistry, Ljubljana, Slovenia

## Abstract

Alternative polyadenylation (APA) determines mRNA stability, localisation, translation and protein function. Several diseases, including obesity, have been linked to APA. Studies have shown that single nucleotide polymorphisms in polyadenylation signals (PAS-SNPs) can influence APA and affect phenotype and disease susceptibility. However, these studies focussed on associations between single PAS-SNP alleles with very large effects and phenotype. Therefore, we performed a genome-wide screening for PAS-SNPs in the polygenic mouse selection lines for fatness and leanness by whole-genome sequencing. The genetic variants identified in the two lines were overlapped with locations of PAS sites obtained from the PolyASite 2.0 database. Expression data for selected genes were extracted from the microarray expression experiment performed on multiple tissue samples. In total, 682 PAS-SNPs were identified within 583 genes involved in various biological processes, including transport, protein modifications and degradation, cell adhesion and immune response. Moreover, 63 of the 583 orthologous genes in human have been previously associated with human diseases, such as nervous system and physical disorders, and immune, endocrine, and metabolic diseases. In both lines, PAS-SNPs have also been identified in genes broadly involved in APA, such as *Polr2c*, *Eif3e* and *Ints11*. Five PAS-SNPs within 5 genes (*Car*, *Col4a1, Itga7*, *Lat*, *Nmnat1*) were prioritised as potential functional variants and could contribute to the phenotypic disparity between the two selection lines. The developed PAS-SNPs catalogue presents a key resource for planning functional studies to uncover the role of PAS-SNPs in APA, disease susceptibility and fat deposition.

**Supplementary Information:**

The online version contains supplementary material available at 10.1007/s00335-022-09967-8.

## Introduction

Various cellular mechanisms determine both the outcome of transcription and the function of the protein encoded by the same gene. Alternative polyadenylation (APA) has recently attracted a great deal of attention (Gebauer and Hentze 2004; Zhang et al. 2021) as it critically affects mRNA stability, localization, translation, protein coding and localization, and is also instrumental in the regulation of gene expression and gene function (Yuan et al. 2021a). It is estimated that more than a third of mouse and two-thirds of human genes undergo alternative polyadenylation events, resulting in various APA transcripts of a single gene (Yuan et al. 2021a). While 80% of APA events occur in the 3′ UTR, resulting in transcripts with different 3′ UTR lengths (Nourse et al. 2020) and consequently with preserved or lost interaction sites for regulators such as miRNAs, lncRNAs and RNA-binding proteins (RBPs) (Tian and Manley 2017), 20% of APA events occur upstream of the last terminal exon, often in an alternatively spliced intron (Nourse et al. 2020). The latter can lead to mRNA decay pathways or the production of truncated proteins (Yuan et al. 2021a).

Cleavage at a site where poly(A) tail is attached to the pre-mRNA (APA site) is regulated by adjacent *cis*-regulatory RNA elements, among which the polyadenylation signal (PAS) motif AAUAAA and its main variant AUUAAA, which are typically located approximately 20-nt upstream of the APA site, are the main components (Shulman and Elkon 2020). These are recognised by cleavage and polyadenylation specificity factors (CPSFs), of which CPSF73 performs the cleavage process (Mandel et al. 2006). Other regulatory sequences and protein complexes include UGUA accessory elements recognised by members of the mammalian cleavage factor Im complex (CFIm), and the GU-/U-rich sequence downstream of APA targeted by cleavage stimulation factors (CSTFs). In addition, the mammalian cleavage factor IIm complex (CFIIm) and the single factors poly(A) polymerase (PAPOL), poly(A)-binding proteins (PABPs) and symplekin scaffold protein (SYMPK) are also required for the cleavage and polyadenylation process (Yuan et al. 2021a).

The important role of APA in cell growth, proliferation and differentiation is well known. However, recent studies have also shown that APA is involved in various abnormal physiological conditions, including endocrine, haematological, oncological, immunological and neurological diseases (Chang et al. 2017). Surprisingly, although both *in vitro* and *in vivo* studies have shown that single nucleotide changes (SNP) in the PAS hexamer or its complete removal severely impair cleavage efficiency (Neve et al. 2017), a limited number of studies have focussed on the relationship between the polymorphic genetic code in PAS and the aetiology of a particular disease. For example, the 3′ UTR variant rs78378222 in *TP53*, which changes the canonical PAS AATAAA to AATACA, increases the risk of various cancers (Wang et al. 2016). The recessive 3′ UTR mutation c.*59A > G in PAS of the gene *INS* leads to reduced mRNA stability and, consequently, neonatal diabetes through reduced insulin biosynthesis (Garin et al. 2010). A mutation in the PAS signal of the BMP1 short transcript decreases its expression and causes bone fragility in children (Fahiminiya et al. 2015), and the PAS mutation (AAUAAA to AAUGAA) in the *FOXP3* gene leads to the IPEX syndrome (Bennett et al. 2001).

Obesity, considered by many a 21st-century epidemic, has been widely believed to result from a disequilibrium between energy intake and expenditure. However, the aetiology of obesity is more complex, resulting from various factors, including genetic predispositions (González-Muniesa et al. 2017). Depending on the genes involved, three types of obesity have been described, of which polygenic obesity, in which a number of genes, each having a small effect, contribute to the phenotype, is the most common clinical manifestation. The other two types are monogenic and syndromic obesities. The latter is associated with mental retardation, dysmorphic features and organ-specific developmental anomalies (Huvenne et al. 2016). Recent studies have shown that APA may also be involved in the development of obesity. For example, targeting the cytoplasmic polyadenylation element-binding protein CPEB4 protects against diet-induced obesity (Pell et al. 2021). Also, in a high-fat-diet-induced obesity rat model, ~89,000 unique alternatively polyadenylated transcripts were identified in the hypothalamus, including those from protein-coding genes, miRNAs and lncRNAs (Brutman et al. 2018), indicating the importance of APA in the regulation of obesity. Moreover, APA events have been associated with tail fat deposition in sheep (Yuan et al. 2021b). Furthermore, while APA-induced truncation of heme oxygenase 1 (HO1) 3′ UTR has a strong inhibitory effect on preadipocyte differentiation of 3T3-L1 cells (Cui et al. 2021), 3′ UTR shortening of the adipogenesis-associated Mth938 domain containing (AAMDC) protein has a positive effect on the differentiation process of bovine preadipocytes (Xiao et al. 2019).

**Fig. 1:**
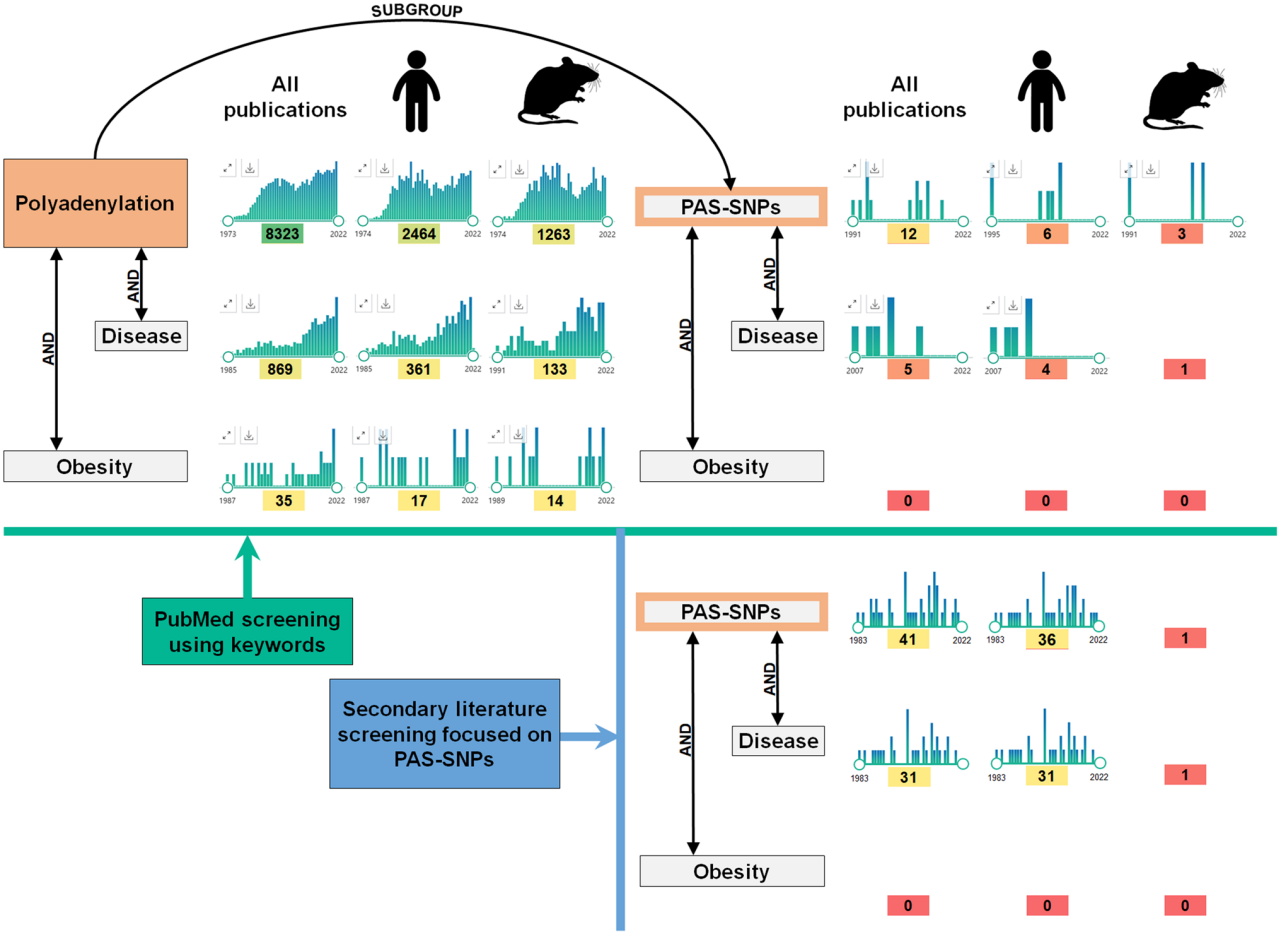
The number of publications extracted from PubMed using keywords related to polyadenylation, PAS-SNPs and their relationship with diseases and obesity, and those found by manual screening. The used keywords are summarized in Supplementary Table S1

Based on the above studies, we hypothesised that DNA polymorphisms in PAS hexamers might be involved in the regulation of APA, ultimately influencing fat deposition and leading to an obese or lean phenotype. However, to our knowledge, no study has yet been conducted to investigate the association between SNPs in PAS (PAS-SNPs) and obesity (Fig. 1).

In the present study, we used unique mouse models established over 60 generations of divergent selection for increased (Fat line—FLI) and decreased (Lean line—FHI) body fat percentage (Sharp et al. 1984) that may best represent the polygenic type of human obesity and leanness (Horvat et al. 2000; Bünger et al. 2001; Simončič et al. 2008, 2011). Apart from using a unique genetic model in which long-term selection fixed opposing obesity or obesity resistance alleles in the Fat and Lean lines, respectively, our study is also novel in terms of the analytical approach. In contrast to a limited number of previous studies in which PAS-SNPs were uncovered by examining single SNP associations between a disease and a PAS-SNP allele with very large effects, our study is designed to systematically search  for genome-wide PAS candidates including alleles with medium and small effects.

To indicate the importance of PAS-SNPs in the development of obesity and to promote this field of research, the aims of the present study were (1) to identify PAS-SNPs in the divergent mouse models for body fat percentage, (2) to analyse the biological processes of genes with PAS-SNPs using bioinformatics databases, (3) to identify disease-, obesity- and APA-related genes with PAS-SNPs and (4) to identify PAS-SNPs within candidate genes with potential functional impact on their expression.

## Materials and methods

### Literature search

Literature related to polyadenylation, PAS-SNPs and their relationship with diseases and obesity was screened on 12/01/2022 in the PubMed database (https://pubmed.ncbi.nlm.nih.gov/) using search terms described in Supplementary Table S1.

### Mouse selection lines

Starting with a base population of the inbred (JU, CBA) and outbred (CFLP) mouse lines, obese (FLI, Fat line) and lean (FHI, Lean line) mouse lines have been established by divergent selection for over sixty generations for increased or decreased body fat percentage (Sharp et al. 1984). The initial body fat of approximately 10% increased to 22% and decreased to 4% in the Fat and Lean lines, respectively, which resulted from the gradual accumulation of “obese” or “lean’’ alleles (Bünger and Hill 1999).

### Identifying SNPs from whole-genome sequencing

The Illumina NextSeq 500 platform was used for the whole-genome sequencing (WGS) of DNA samples from the Fat and Lean mouse lines. Sequencing reads were first preprocessed according to the FastQC report and then mapped to the mouse reference genome (version GRCm38.86) using the Burrows-Wheeler Aligner (BWA) alignment tool (Li and Durbin 2009). Base quality score recalibration, indel realignment, duplicate removal, variant calling and hard filtering were performed according to the Genome Analysis Toolkit (GATK) (McKenna et al. 2010) Best Practices recommendations (Depristo et al. 2011; Van der Auwera et al. 2013). Variants were annotated using the Ensembl Variant Effect Predictor (https://www.ensembl.org/Tools/VEP) (McLaren et al. 2016).

### Identification of SNPs overlapping PAS motif

The locations of SNPs identified by WGS that differ between the lines were overlapped with locations of PAS motifs obtained from the PolyASite 2.0 portal (https://polyasite.unibas.ch/) (Herrmann et al. 2020), considering strand-specific PAS and APA genomic positions to identify the genes with PAS-SNPs. An example is provided in Supplementary Fig. S1. Only PAS motifs within genes were included in the analysis.

### Identification of SNPs overlapping PAS motif in obesity- and polyadenylation-related genes

Obesity-related genes were obtained from the literature (Mlinar et al. 2006; Swami 2009; Rao et al. 2014; da Fonseca et al. 2017; D’Angelo et al. 2018; Flores-Dorantes et al. 2020; Agrawal et al. 2021; Loos and Yeo 2022). In total, the list contains 431 genes (Supplementary Table S2). Genes (281) associated with polyadenylation were retrieved from various databases: AmiGO 2 (http://amigo.geneontology.org/amigo), Reactome (https://reactome.org/), QuickGO (https://www.ebi.ac.uk/QuickGO/annotations), KEGG (https://www.kegg.jp/), and Mouse Genome Informatics (http://www.informatics.jax.org). A set of genes is given in Supplementary Table S3.

### In silico functional analysis

To get a potential functional biological impact of PAS-SNPs, first, GO enrichment analysis of PAS-SNP containing genes was done using MonaGO (https://monago.erc.monash.edu/) (Xin et al. 2020), a visualization tool that uses GO annotation information from DAVID (https://david.ncifcrf.gov/home.jsp).

Disease-associated genes were identified using Mouse Genome Informatics (http://www.informatics.jax.org), and disease–gene interactions were visualized in Cytoscape (Version 3.9.0) (Shannon 2003). The miRNA target sites were obtained from the miRTarBase (Huang et al. 2019).

Multiple-species alignment of the genes with PAS-SNPs was performed using the Ensembl alignment tool (Howe et al. 2021). For the human SNPs at orthologous positions, their location relative to known APA sites and PAS motifs was analysed using the PolyASite 2.0 portal (https://polyasite.unibas.ch/) (Herrmann et al. 2020).

### Expression of selected genes using microarray and prioritisation of candidate PAS-SNPs

To examine the expression of common (genes containing PAS-SNPs in both lines), disease-associated, and obesity- and APA-related genes carrying PAS-SNPs identified in either line, the expression data were obtained from the microarray transcriptome profiling performed on various mouse tissues, including white adipose tissue (WAT) [subcutaneous (sWAT), epididymal (eWAT), and mesenteric (mWAT)], brown adipose tissue (BAT), liver, muscle, adrenal gland, thymus and kidney. After RNA preparation, samples were hybridized to the Affymetrix Mouse Genome 430–2.0 GeneChip. The obtained data were processed as described previously (Morton et al. 2011; Pedroni et al. 2014). The expression of the genes was considered differential when the expression between Fat and Lean mouse lines differed by at least 1.5-fold at *p* < 0.05 (DEGs). The *p* value rather than adjusted *p* value was used to avoid losing the potential candidate PAS-SNPs; however, for the DEGs carrying candidate PAS-SNPs, their expression was also checked at the adjusted *p* < 0.05. The expressions of differentially expressed disease-related, common, obesity-related and APA-related genes carrying PAS-SNPs are given in Supplementary table S4.

Then, Affymetrix probes (locations were obtained from the Ensembl database) of DEGs were along with PAS-SNPs mapped to their corresponding genes using the Golden Helix GenomeBrowse® v3.0.0 visualization tool (http://www.goldenhelix.com) (Golden Helix, Inc, Bozeman, MT) to identify candidate PAS-SNPs. The PAS-SNPs were prioritised considering their effect on PAS and locations relative to the Affymetrix probes and their expressions. For the candidate PAS-SNPs, the DNA sequence within the APA cluster and 60 bp upstream was analysed to explore whether other PAS are present and whether other SNPs in the two lines may create de-novo PAS of the corresponding APA site.

## Results

**Fig. 2:**
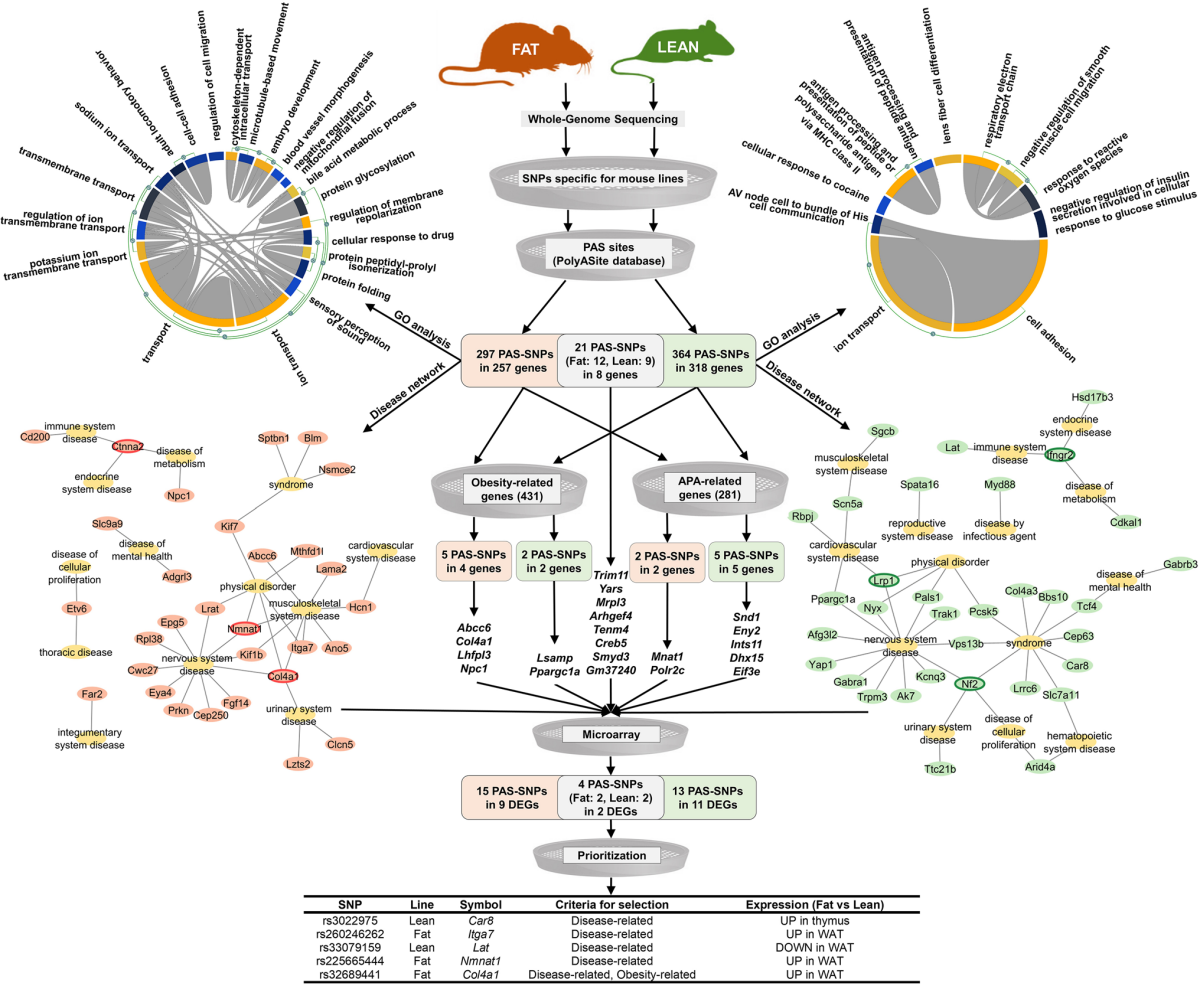
Workflow and main results. Steps include Genotyping of Fat and Lean mouse selection lines, identification of PAS-SNPs, GO enrichment analysis by MonaGO, identification of disease-related genes (MGI) and construction of gene-disease interaction network visualized by Cytoscape, identification of common (genes containing PAS-SNPs in both lines), and obesity and APA-related genes having PAS-SNPs identified in Fat or Lean line

The PolyASite 2.0 database [Release Mus musculus: v2.0 (GRCm38.96)] describes 301,006 APA sites in the mouse genome, with the AATAAA PAS motif being the most abundant, followed by ATTAAA (Supplementary Fig. S2). In the present study, we identified whole-genome PAS-SNPs specific for the Fat (309) and Lean (373) mouse selection lines for body fat percentage, located in 265 and 326 genes. Details (including PAS-SNPs identified in both lines) of PAS-SNPs are found in Supplementary Table S5. The GO enrichment analysis revealed that these genes are involved in various biological processes, but in both lines they predominantly participate in cellular transport. The two lines share 8 common genes containing PAS-SNPs that affect different APA sites within a single gene. Also, 30 (Fat line) and 33 (Lean line) genes have already been linked to human diseases. A large proportion of these genes in both lines are related to the nervous system (Fat: 36.7%, Lean: 39.4%) and physical disorders (Fat: 20.0%, Lean: 12.1%). Moreover, in the Fat line, several genes are involved in musculoskeletal disorders (Fat: 26.7%), while in the Lean line they are implicated in various syndromes (Lean: 30.3%). Furthermore, a group of genes participating in immune, endocrine and metabolic diseases were identified in both lines. In addition, a total of 14 PAS-SNPs within 6 obesity- and 7 APA-related genes were identified. 22 candidate genes carrying PAS-SNPs were differentially expressed between the two lines. Finally, manual examination prioritised 5 PAS-SNPs within *Car8*, *Itga7*, *Lat*, *Nmnat1* and *Col4a1*. The workflow and main results are shown in Fig. 2.

### Genotyping

Genotyping revealed that 309 and 373 SNPs specific for either the Fat or Lean line are located in PAS within 265 and 326 genes. Interestingly, only 8 of the total 583 genes are shared between the two lines, carrying PAS-SNPs at different APA sites within the genes (*Trim11*, *Yars*, *Mrpl3*, *Arhgef4*, *Tenm4*, *Creb5*, *Smyd3*, *Gm37240*) (Supplementary Fig. S3).

GO enrichment analysis revealed that genes with PAS-SNPs are involved in various biological processes (BP), some of them even in several BP. In both lines, a large number of these genes participate in cellular transport. Moreover, the genes in the Fat line also play a role in cytoskeletal organization and in protein modifications (protein glycosylation, protein peptidyl-prolyl isomerization). Meanwhile, in the Lean line, they participate in cell adhesion, cellular respiration and response to stimuli (Supplementary Fig. S4).

To investigate whether the sequence variants identified in the Fat and Lean mouse lines are mouse-specific or whether SNPs exist at orthologous positions in other species, we performed cross species sequence alignment using the Ensemble alignment tool. 32 PAS-SNPs are located at orthologous positions in other species (Table 1).

**Table 1:** SNPs identified in the Fat and Lean mouse lines at orthologous positions of sequence variants in other species

Mouse		Other species				Human	
Variant ID	Gene symbol	Species	Variant ID	Variant type	Gene symbol	APA ID (PolyASite)	Variant location relative to PAS and APA (PolyASite)
rs36810866	*A830018L16Rik*	Human	rs574671612	SNP	*C8orf34* (ortholog of *A830018L16Rik*)		
rs30860267	*Kdm5b*	Human	rs921113807	SNP	*KDM5B*		
rs250253373	*Dner*	Human	rs1283834778	SNP	*DNER*		
rs30937553	*Gm40770*	Human	rs918141202	SNP	/		
rs52039424	*1700025F24Rik*	Human	rs887567006	SNP	Upstream of ENSG00000257083		
rs218957174	*Mthfd1l*	Human	rs1360996180	SNP	*MTHFD1L*		
rs52557469	*Abca5*	Human	rs1281346561	SNP	*ABCA5*	17:69,247,483:+	Upstream of known PAS (45 bp from APA) in antisense direction
rs224932127	*Asic2*	Human	rs1400720214	SNP	*ASIC2*		
rs48386684	*Wfikkn2*	Human	rs1017487641	SNP	*WFIKKN2*	17:50,842,346:+	Between 2 PAS
rs4208259	*Cxadr*	Human	rs943743662	SNP	*CXADR*	21:17,569,608 +	Between 2 PAS
rs1132123542	*Snx24*	Human	rs557040637	SNP	*SNX24*	5:123,009,226:+	In PAS
rs47475774	*Ccna2*	Human	rs755179571	SNP	*CCNA2*		
rs250712171	*Gm45604*	Pig	rs703127080	SNP	*SLC16A7*		
rs26901910	*Nf2*	Sheep	rs599786180	SNP	*NF2*		
rs51031107	*1700060O08Rik*	Horse	rs68603952	SNP	/		
rs30155538	*Mcur1*	Horse	rs782875901	SNP	Downstream of *MCUR1*		
rs243180722	*Lsamp*	Goat	rs671877994	SNP	/		
rs30463516	*Tspan2os*	Human	rs1169250871	SNP	/		
rs51812848	*Grid1*	Human	rs111881471	indel	*GRID1*		
rs32419944	*Slc38a2*	Human	rs1351333839	indel	*SLC38A2*	12:46,360,463:−	Over part of PAS
rs29870198	*Fbxo38*	Human	rs1422927536	indel	*FBXO38*	5:148,442,330:+	Over parts of 2 PAS
rs27462803	*Ckap2l*	Human	rs943325762	indel	*CKAP2L*		
rs30152485	*Wdr3*	Human	rs754569327	indel	*WDR3*	1:117,959,396:+	Within APA cluster
			rs1192320348	indel			
rs31269486	*Gm36569*	Human	rs1232100051	indel	*GASK1B* and *GASK1B-AS1*		
rs3022975	*Car8*	Human	rs1208110829	indel	*CA8*	8:60,189,629:−	Upstream of known PAS (31 bp from APA)
rs37020130	*Dhx15*	Human	rs1027076262	indel	*DHX15*	4:24,539,852:−	Within APA cluster
rs585906802	*Ctnna2*	Human	rs546967032	indel	*CTNNA2*		
rs52130811	*Slc35f5*	Human	rs554688324	indel	*SLC35F5*		
rs32088144	*Brinp3*	Human	rs1343625029	indel	*BRINP3*		
rs213215053	*Col4a3*	Human	rs1457057611	indel	*COL4A3*		
rs244556382	*5730522E02Rik*	Human	rs1464977908	indel	*LINC01122*		
rs231153192	*Ggh*	Human	rs111869558	indel	*GGH*		
			rs1421773187	indel			
			rs1429142810	indel			
			rs1474924870	indel			
			rs898026262	deletion			

**Fig. 3:**
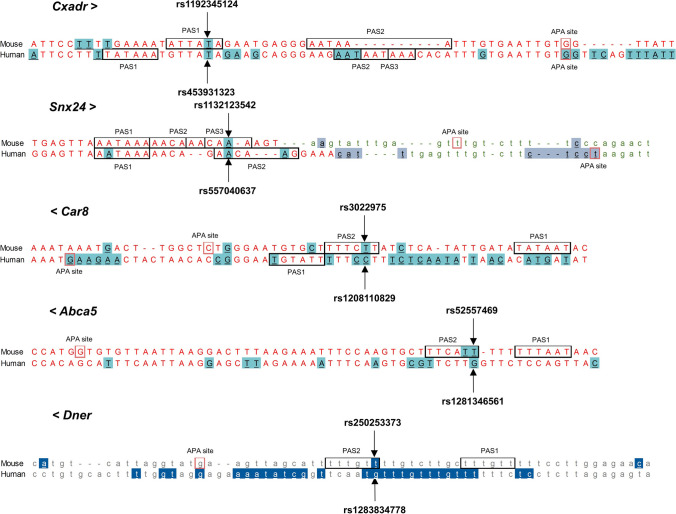
Multiple-species alignment of parts of the selected genes with PAS-SNPs with marked genetic variants, PAS motifs, and APA sites. Legend: capital red letters—nucleotides in 3′ UTR, capital black letters shaded light blue—3′ UTR variants, small green letters—nucleotides downstream of the gene, small black letters shaded grey—downstream variants, small grey letters—nucleotides in the intron, small white letters shaded blue—intronic variants

Of note, PAS-SNP rs1132123542 in *Snx24* of the Fat line is located at the orthologous position of human PAS-SNP rs943743662. Interestingly, the *Snx24* transcripts in both species (mouse and human) may be longer than annotated, as suggested by the APA sites located downstream of the genes. Alignment examples of parts of the mouse and human *Cxadr*, *Snx24*, *Car8*, *Abca5* and *Dner* are given in Fig. 3.

### Disease-related genes with PAS-SNPs

**Fig. 4:**
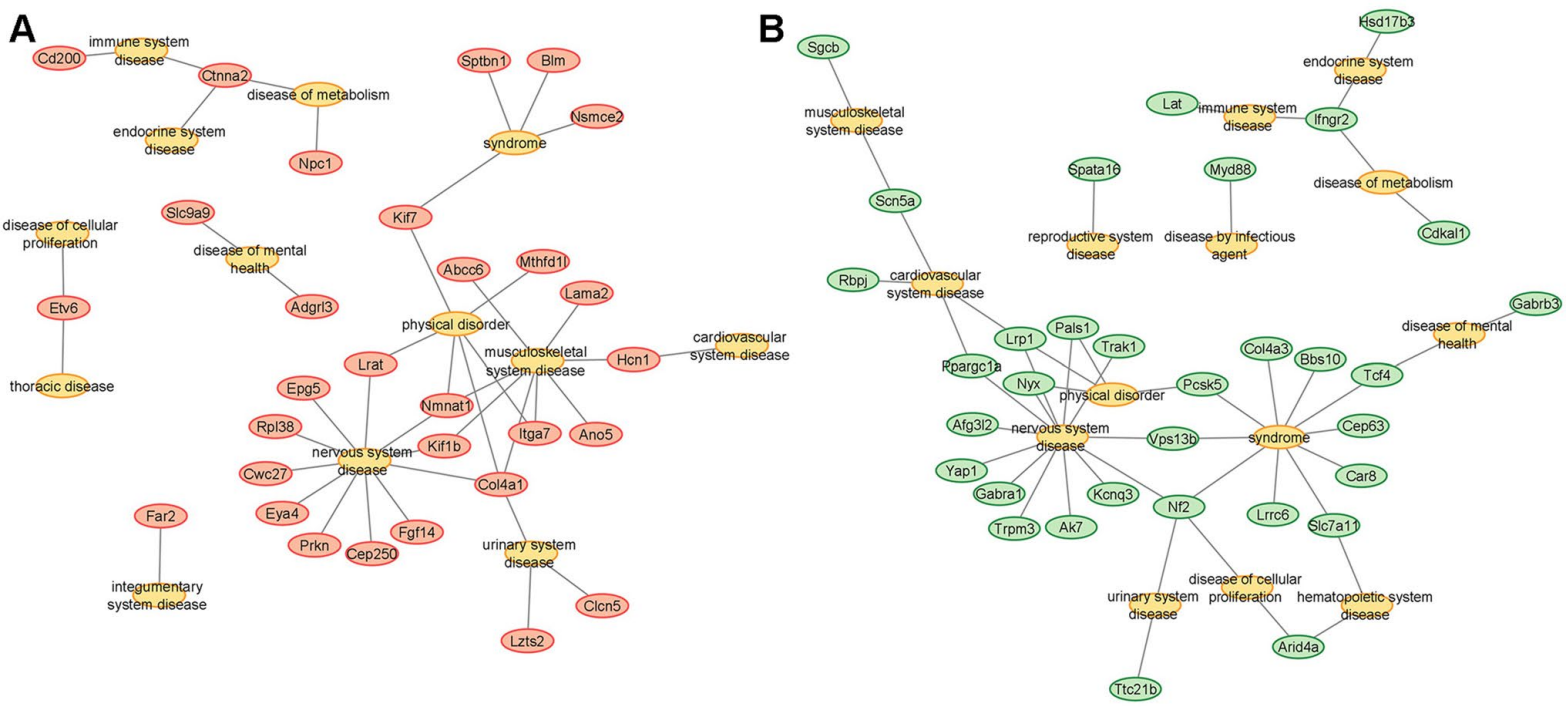
Disease network of genes with PAS-SNPs identified in Fat **A** and Lean **B** mouse selection lines for body fat percentage

30 (Fat line) and 33 (Lean line) human orthologous genes have already been associated with diseases. A large percentage of these genes are linked with nervous system and physical disorders in both lines. Moreover, several genes are involved in musculoskeletal disorders in the Fat line, while they are associated with syndromic diseases in the Lean line. Furthermore, a cluster of genes participating in immune, endocrine and metabolic diseases have been identified in both lines. Considering the involvement of a particular gene in various diseases, *Col4a1* (linking nervous, musculoskeletal and urinary system diseases and physical disorders), *Ctnna2* (linking immune, endocrine, and metabolic diseases), and *Nmnat1* (linking nervous and musculoskeletal diseases and physical disorders) might be core genes containing PAS-SNPs in the Fat line (Fig. 4A). Meanwhile, the core genes in the Lean line might be *Nf2* (linking syndromic, nervous, urinary and cellular proliferative diseases), *Lrp1* (linking cardiovascular and nervous system diseases and physical disorders), and *Ifngr2* (linking immune, endocrine and metabolic diseases) (Fig. 4B).

### Common genes with PAS-SNPs in Fat and Lean lines

There are eight common genes with PAS-SNPs identified in both lines but influencing different APA sites: *Trim11*, *Yars*, *Mrpl3*, *Arhgef4*, *Tenm4*, *Creb5*, *Smyd3*, *Gm37240*. The location of the SNPs and APA sites with the corresponding PAS hexamer within the eight genes is given in Table 2.

**Table 2:** Location, APA site, PAS hexamer and its change due to the SNP within common genes of Fat and Lean lines

Variant				Polyadenylation region				
Mouse line	Variant ID	Location	Gene symbol	APA ID	Type	PAS	PAS with alternative allele	Consequence on PAS
Fat	rs252244088	11:58,980,172	*Trim11*	11:58,980,198:+	IN	AATA**A**A	AATA**C**A	MA → LA motif
Lean	rs29473166	11:58,979,030	*Trim11*	11:58,979,039:+	IN	T**A**TAAA	T**G**TAAA	Loss
Fat	rs27517013	4:129,219,177	*Yars*	4:129,219,188:+	TE	CATAA**A**	CATAA**G**	Loss
Lean	rs3167104	4:129,197,453	*Yars*	4:129,197,470:+	IN	AATA**T**A	AATA**C**A	MA → LA motif
Fat	rs259336677	9:105,077,523	*Mrpl3*	9:105,077,566:+	TE	AAGAA**A**	AAGAA**G**	Loss
Fat	rs238373229	9:105,077,540	*Mrpl3*	9:105,077,566:+	TE	AATA**T**A	AATA**A**A	LA → MA motif
Lean	rs50804946	9:105,077,439	*Mrpl3*	9:105,077,462:+	TE	TATAA**A**	TATAA**G**	Loss
Fat	rs581302908	1:34,809,203	*Arhgef4*	1:34,809,218:+	TE	AATA**A**A	AATA**G**A	MA → LA motif
Lean	rs46757124	1:34,691,617	*Arhgef4*	1:34,691,633:+	IN	A**A**TAAA	A**G**TAAA	MA → LA motif
Fat	rs13459552	7:96,910,302	*Tenm4*	7:96,910,339:+	TE	A**G**TAAA	A**A**TAAA	LA → MA motif
Lean	rs51720083	7:96,237,422	*Tenm4*	7:96,237,473:+	IN	AT**T**AAA	AT**C**AAA	Loss
Fat	rs29969996	6:53,639,550	*Creb5*	6:53,639,571:+	IN	AAT**G**AA	AAT**A**AA	LA → MA motif
Lean	rs29943631	6:53,297,572	*Creb5*	6:53,297,588:+	IN	ATTAA**A**	ATTAA**C**	Loss
Fat	rs220155151	1:178,956,075	*Smyd3*	1:178,956,062:−	TE	AATA**C**A	AATA**T**A	LA → MA motif
Fat	rs241932144	1:178,957,273	*Smyd3*	1:178,957,259:−	TE	AT**T**AAA	AT**A**AAA	Loss
Fat	rs236503723	1:179,168,920	*Smyd3*	1:179,168,907:−	IN	ATTA**A**A	ATTA**T**A	MA → LA motif
Fat	rs235763389	1:179,508,325	*Smyd3*	1:179,508,286:−	TE	ATT**A**AA	ATT**T**AA	Loss
Lean	rs48210465	1:179,394,422	*Smyd3*	1:179,394,400:−	IN	AATA**C**A	AATA**T**A	LA → MA motif
Fat	rs30706788	3:84,809,818	*Gm37240*	3:84,809,806:−	IN	**A**TTAAA	**G**TTAAA	Loss
Lean	rs258005769	3:85,755,470	*Gm37240*	3:85,755,450:−	IN	A**G**TAAA	A**A**TAAA	LA → MA motif
Lean	rs30399416	3:85,789,619	*Gm37240*	3:85,789,600:−	IN	AC**T**AAA	AC**C**AAA	Loss

Legend: column Type, *IN* intronic APA site, *TE* terminal exon APA site; column Consequence, *MA* more abundant, *LA* less abundant

Moreover, not only are PAS-SNPs located in the two lines in PAS of different APAs within a given gene, PAS-SNPs also alter the PAS sites to more or less abundant PAS hexamers or cause their complete loss according to the previously annotated PAS (Supplementary Fig. S2). For example, in the *Tenm4* gene, a PAS-SNP rs13459552 of the Fat line located in the 3′ UTR alters the AGTAAA to a more important canonical AATAAA. Meanwhile, in the Lean line, an intronic PAS-SNP rs51720083 causes a complete loss of PAS (Supplementary Fig. S5).

Another interesting example is *Mrpl3*. While the SNP rs259336677 in the Fat line causes loss in PAS (AAGAAA > AAGAAG), the SNP rs238373229 alters AATATA to the canonical AATAAA of the APA site 9:105,077,566: + . Meanwhile, in the Lean line, SNP rs50804946 causes loss of one of the two PASs (TATAAA > TATAAG) of the APA site 9:105,077,462: + , which is the most frequently observed APA site of *Mrpl3*, occurring in 79.5% of *Mrpl3* alternative polyadenylation events, according to PolyASite (Supplementary Fig. S6).

**Fig. 5:**
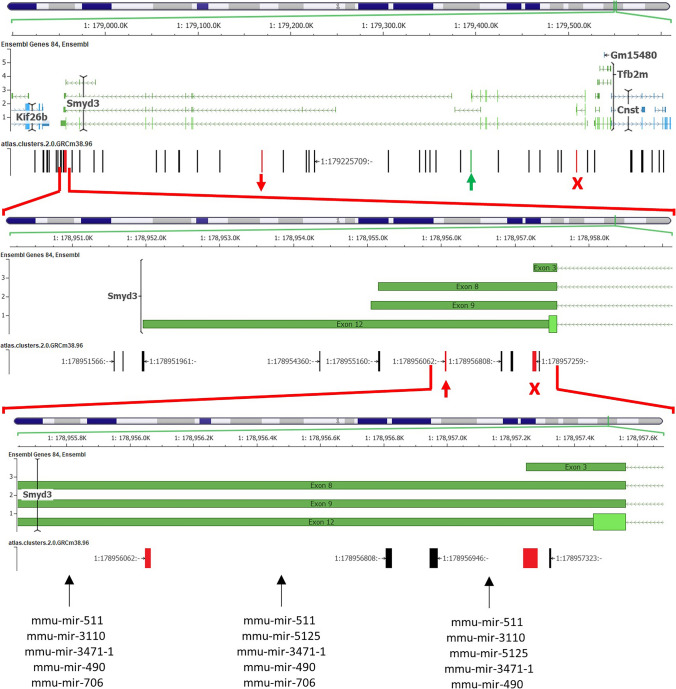
PAS-SNPs within *Smyd3*. ↑—altered PAS hexamer to a more used PAS, ↓—altered PAS hexamer to a more used PAS, X—lost PAS by SNP. Red—Fat line, Green—Lean line. Approximate miRNA target locations are also indicated (miRTarBase—predicted by miRanda)

Even more PAS-SNPs were identified in *Smyd3* (Fat: 4, Lean: 1). Interestingly, in the Fat line, SNP rs241932144 in 3′ UTR causes loss of PAS (ATTAAA > ATAAAA) of mostly used APA site (1:178,957,259:−) within *Smyd3*, occurring in 86.3% of alternative polyadenylation events (PolyASite). Meanwhile, the SNP rs220155151 alters a less used PAS AATACA to a more used AATATA of APA site located downstream (1:178,956,062:−). At the same time, other SNPs detected in the Fat line alter PAS to a less used hexamer or cause its loss. Changes in PAS hexamers in 3′ UTR may influence the length of the *Smyd3* transcripts and consequently affect miRNA binding (Fig. 5).

### Obesity-related genes with PAS-SNPs

We then selected 431 genes that are associated with obesity and identified 5 PAS-SNPs (rs32753534, rs32689441, rs47853609, rs48552886, rs587469149) in 4 obesity-related genes in the Fat line (*Abcc6*, *Col4a1*, *Lhfpl3*, *Npc1*) and 2 PAS-SNPs (rs243180722, rs38383450) in 2 obesity-related genes in the Lean line (*Lsamp*, *Ppargc1a*). All the SNPs are intronic variants of their corresponding genes. Considering the representation and usage of PAS motifs in the mouse genome described in the Supplementary Fig. S2, rs32753534 and rs38383450 change the PAS to a more used PAS hexamer. Meanwhile, the opposite was found for the remaining PAS-SNPs (rs32689441, rs47853609, rs48552886, rs587469149, rs243180722) that cause a complete loss of previously identified PAS motifs (Table 3).

**Table 3:** Location, APA site, PAS hexamer and its change due to the SNP within obesity-related genes of Fat and Lean lines

Variant				Polyadenylation region				
Mouse line	Variant ID	Location	Gene symbol	APA ID	Type	PAS	PAS with alternative allele	Consequence on PAS
Fat	rs32753534	7:45,971,020	*Abcc6*	7:45,970,994:−	IN	ATTA**C**A	ATTA**A**A	LA → MA motif
Fat	rs32689441	8:11,302,646	*Col4a1*	8:11,302,636:−	IN	AAG**A**AA	AAG**G**AA	Loss
Fat	rs47853609	5:22,926,791	*Lhfpl3*	5:22,926,806:+	IN	AATG**A**A	AATG**G**A	Loss
Fat	rs48552886	5:22,926,797	*Lhfpl3*	5:22,926,806:+	IN	GAT**A**AA	GAT**G**AA	Loss
Fat	rs587469149	18:12,210,261	*Npc1*	18:12,210,214:−	IN	**A**ATAGA	**T**ATAGA	Loss
Lean	rs243180722	16:41,720,764	*Lsamp*	16:41,720,799:+	IN	AATAT**A**	AATAT**T**	Loss
Lean	rs38383450	5:51,561,938	*Ppargc1a*	5:51,561,914:−	IN	**T**ATAAA	**A**ATAAA	LA → MA motif

Legend: column Type, *IN* intronic APA site, *TE* terminal exon APA site; column Consequence, *MA* more abundant, *LA* less abundant

An example of PAS-SNP rs38383450 (T > A) in the first intron of *Ppargc1a* transcript ENSMUST00000127135 identified in the Lean line is given in Supplementary Fig. S7. The SNP changes TATAAA PAS hexamer to the canonical AATAAA.

### APA-related genes with PAS-SNPs

We then selected 281 genes that are involved in various aspects of alternative polyadenylation. 2 SNPs (rs29182020, rs579356626) in the Fat line are in PAS of two APA-related genes (*Mnat1*, *Polr2c*), and 5 SNPs (rs33836529, rs33289253, rs227466545, rs37020130, rs33250559) in the Lean line are in PAS of 5 APA-related genes (*Snd1*, *Eny2*, *Ints11*, *Dhx15*, *Eif3e*). While rs579356626, rs33289253 and rs227466545 are 3′ UTR variants, the remaining four SNPs are in the introns of the corresponding genes. Considering the representation and usage of PAS motifs in the mouse genome described in Supplementary Fig. S2, rs579356626, rs33836529, rs227466545 and rs37020130 change the most used PAS to a less used PAS hexamer. Meanwhile, rs29182020, rs33289253 and rs33250559 cause a complete loss of previously identified PAS motifs (Table 4).

**Table 4:** Location, APA site, PAS hexamer and its change due to the SNP within obesity-related genes of Fat and Lean lines

Variant				Polyadenylation region				
Mouse line	Variant ID	Location	Gene symbol	APA ID	Type	PAS	PAS with alternative allele	Consequence on PAS
Fat	rs29182020	12:73,176,649	*Mnat1*	12:73,176,668:+	IN	AAT**A**AA	AAT**T**AA	Loss
Fat	rs579356626	8:94,873,520	*Polr2c*	8:94,873,526:+	TE	AATAA**A**	AATAA**G**	MA → LA motif
Lean	rs33836529	6:28,799,697	*Snd1*	6:28,799,710:+	IN	AATA**A**A	AATA**C**A	MA → LA motif
Lean	rs33289253	15:44,436,556	*Eny2*	15:44,436,573:+	TE	AATA**A**T	AATA**T**T	Loss
Lean	rs227466545	4:155,889,082	*Ints11*	4:155,889,098:+	TE	AATA**A**A	AATA**C**A	MA → LA motif
Lean	rs37020130	5:52,159,959	*Dhx15*	5:52,159,940:−	IN	A**A**TAAA	A**G**TAAA	MA → LA motif
Lean	rs33250559	15:43,268,200	*Eif3e*	15:43,268,181:−	IN	AGT**A**AA	AGT**G**AA	Loss

Legend: column Type, *IN* intronic APA site, *TE* terminal exon APA site; column Consequence, *MA* more abundant, *LA* less abundant

An example of PAS-SNP rs33289253 (A > T) in 3′ UTR of *Eny2* identified in the Lean line is given in Supplementary Fig. S8. The SNP overlaps with AATAAT PAS hexamer, resulting in lost PAS.

### Expression of selected genes using microarray

In order to identify the PAS-SNPs that may influence the alternative polyadenylation and thus the expression and variability of gene transcripts, we examined the expression of candidate genes with PAS-SNPs (Disease-related: 63, Common: 8, Obesity-related: 6, APA-related: 7) using microarray analyses of various tissues. Note that *Col4a1* is found in both groups: as a core gene in the disease-related network and as an obesity-related gene. Out of 83 candidate genes with PAS-SNPs, 22 genes were differentially expressed between the Fat and Lean lines: *Car8*, *Cdkal1*, *Col4a1*, *Creb5*, *Ctnna2*, *Eny2*, *Fgf14*, *Gabrb3*, *Ifngr2*, *Itga7*, *Kif1b*, *Lat*, *Lhfpl3*, *Nmnat1*, *Pcsk5*, *Ppargc1a*, *Sgcb*, *Slc9a9*, *Sptbn1*, *Tcf4*, *Tenm4* and *Trak1* (Table 5).

**Table 5:** Differentially expressed candidate genes between Fat and Lean lines carrying PAS-SNPs

Variant		Gene			
Variant ID	Mouse line	Gene symbol	Gene name	Criteria for gene selection	Expression (Fat vs Lean)
rs3022975	Lean	*Car8*	carbonic anhydrase 8	Disease-related^1^	UP in thymus*
rs264180954	Lean	*Cdkal1*	CDK5 regulatory subunit associated protein 1-like 1	Disease-related^2^	UP in adrenal gland*, kidney*, thymus*, BAT*, WAT*, sWAT*, mWAT*, eWAT*
rs585906802	Fat	*Ctnna2*	catenin (cadherin associated protein), alpha 2	Disease-related^2,3,4^	UP in WAT, sWAT*, eWAT*
rs46962113, rs234950047, 14_124175608_T/A	Fat	*Fgf14*	fibroblast growth factor 14	Disease-related^5^	DOWN in thymus*, BAT*, sWAT*
rs45740960, rs47131593	Lean	*Gabrb3*	gamma-aminobutyric acid (GABA) A receptor, subunit beta 3	Disease-related^5^	DOWN in liver*
16_91565577_A/T	Lean	*Ifngr2*	interferon gamma receptor 2	Disease-related^2,3,4^	UP in WAT
rs260246262	Fat	*Itga7*	integrin alpha 7	Disease-related^7,8^	UP in WAT*
rs32587514	Fat	*Kif1b*	kinesin family member 1B	Disease-related^5,7^	UP in WAT*
rs33079159	Lean	*Lat*	linker for activation of T cells	Disease-related^3^	DOWN in WAT
rs225665444	Fat	*Nmnat1*	nicotinamide nucleotide adenylyltransferase 1	Disease-related^3,7,8^	UP in WAT
rs36933769	Lean	*Pcsk5*	proprotein convertase subtilisin/kexin type 5	Disease-related^1,8^	UP in adrenal gland*
rs38392118	Lean	*Sgcb*	sarcoglycan, beta (dystrophin-associated glycoprotein)	Disease-related^7^	DOWN in kidney* and BAT*
rs36243838, rs30381123, rs234371181	Fat	*Slc9a9*	solute carrier family 9 (sodium/hydrogen exchanger), member 9	Disease-related^6^	DOWN in WAT
rs13473736, rs262769988	Fat	*Sptbn1*	spectrin beta, non-erythrocytic 1	Disease-related^1^	UP* and DOWN in WAT*, UP in mWAT* and sWAT*, DOWN in kidney*
rs29580819	Lean	*Tcf4*	transcription factor 4	Disease-related^1,6^	UP in WAT
rs29593481, rs219766009	Lean	*Trak1*	trafficking protein, kinesin binding 1	Disease-related^5^	UP in WAT*
rs32689441	Fat	*Col4a1*	collagen type IV alpha 1	Disease-related^5,7,8,9^, Obesity-related	UP in WAT
rs47853609, rs48552886	Fat	*Lhfpl3*	lipoma HMGIC fusion partner-like 3	Obesity-related	UP in adrenal gland*
rs38383450	Lean	*Ppargc1a*	peroxisome proliferative activated receptor, gamma, coactivator 1-alpha	Obesity-related	DOWN in adrenal gland*
rs29969996, rs29943631	Fat, Lean	*Creb5*	cAMP responsive element-binding protein 5	Common	DOWN in adrenal gland*
rs13459552, rs51720083	Fat, Lean	*Tenm4*	teneurin transmembrane protein 4	Common	UP in WAT* and mWAT*, DOWN in adrenal gland*
rs33289253	Lean	*Eny2*	ENY2 transcription and export complex 2 subunit	APA-related	UP in mWAT*

Legend: column Criteria for gene selection,^1^syndrome, ^2^disease of metabolism, ^3^immune system disease, ^4^endocrine system disease, ^5^nervous system disease, ^6^disease of mental health, ^7^musculoskeletal system disease, ^8^physical disorder, ^9^urinary system disease; column Expression (Fat vs Lean), UP—higher expression of Affymetrix probe(s) in the Fat compared to the Lean line, DOWN—lower expression of Affymetrix probe(s) in the Fat compared to the Lean line, *DEG also at adjusted *p* < 0.05

### Prioritisation of candidate PAS-SNPs

**Fig. 6:**
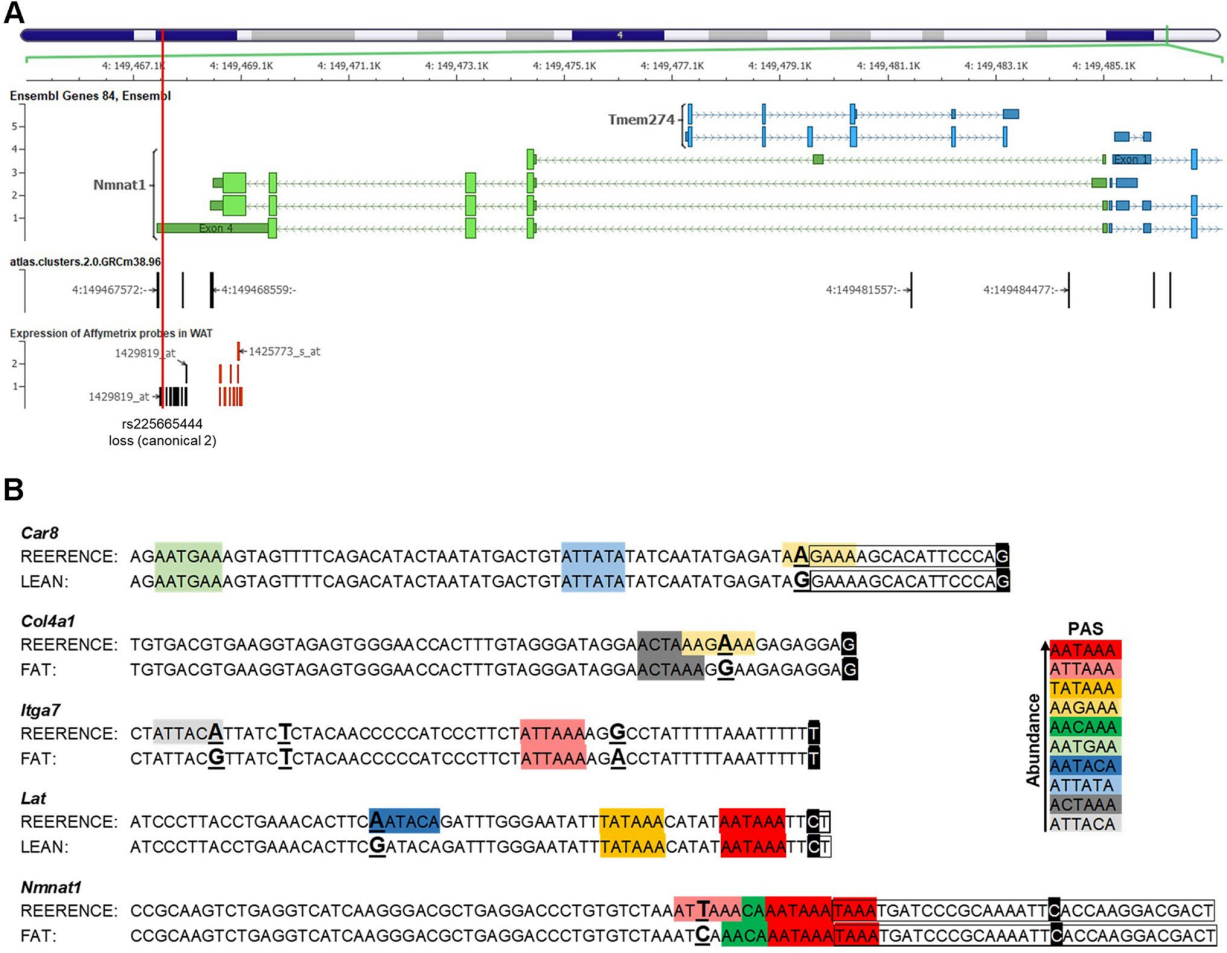
Visual identification of potentially functional PAS-SNPs. **A** Location of PAS-SNPs, Affymetrix probes and their expressions within *Nmnat1*. 3rd track, black rectangles—Affymetrix probes with no expression difference between the lines, red rectangles – the expression being higher in the Fat line compared to the Lean line, red vertical line denotes PAS-SNPs identified in the Fat line. **B** Location of SNPs, PAS motifs and APA sites. Legend: coloured 6 bp hexamers—PAS motifs (the colour denotes their abundance in mouse genome), bigger underlined nucleotides in bold—locations of SNPs, nucleotides coloured black and written white—APA site ID (most often cleavage site in this region according to PolyASite 2.0 portal), nucleotides in a black box—APA cluster (bigger the box more variable the cleavage site)

Affymetrix probes from differentially expressed genes were then mapped to the corresponding genes along with PAS-SNPs to identify candidate PAS-SNPs. Among the 22 DEGs carrying PAS-SNPs, PAS-SNPs of five genes were prioritised, three within *Col4a1*, *Itga7* and *Nmnat1* of the Fat line and two within *Car8* and *Lat* of the Lean line. PAS-SNPs within *Col4a1*, *Itga7* and *Lat* may affect the protein lengths encoded by these genes. Meanwhile, PAS-SNPs rs3022975 and rs225665444 within *Car8* and *Nmnat1* may result in the genes having different 3′ UTR lengths in the two lines (Fig. 6A and Supplementary Fig. S9).

For the five genes, the DNA sequences within the APA cluster and 60 bp upstream were analysed to explore whether other PAS are present and if other SNPs in the two lines may create de-novo PAS of the corresponding APA site. The rs260246262 and rs33079159 within *Itga7* and *Lat* of the Fat and Lean line cause loss of less abundant motifs, whereas the canonical AATAAA and ATTAAA are still present. Similarly, rs225665444 within *Nmnat1* of the Fat line damages the second most important PAS, ATTAAA, but two canonical motifs remain untouched. In contrast, the rs3022975 within Lean line *Car8* and rs32689441 within Fat line *Col4a1* disturb the most abundant signals in the examined regions (Fig. 6B).

### Discussion

Alternative polyadenylation (APA) has recently been established as one of the key mechanisms regulating information transfer from the genome to the phenome (Zhang et al. 2021). APA is regulated by various protein complexes that recognise *cis*-regulatory elements, among which the polyadenylation signal (PAS) is the most important component (Shulman and Elkon 2020). Studies have shown that alterations in PAS can affect the cleavage efficiency, resulting in mRNA transcripts of different lengths and thus different coding potential and proteins with altered functionality (Gebauer and Hentze 2004; Zhang et al. 2021). However, few studies have focussed on whole-genome single nucleotide changes in PAS (PAS-SNPs) and their possible association with disease susceptibility and phenotype.

In the present study, genome-wide identification of PAS-SNPs in two divergent mouse selection lines for body fat percentage was performed as an indication of association with their phenotypic divergence. A total of 309 and 373 PAS-SNPs specific to either the Fat or Lean line were identified within 297 and 325 genes involved in various biological pathways. Pathways of cell adhesion, antigen processing, immune response, and bile acid and glucose metabolism were previously enriched in transcriptome analysis of the two mouse lines (Morton et al. 2011; Simončič et al. 2011). The other large portion of the genes with PAS-SNPs is involved in the intra- and inter-cellular transport of ions and organic compounds. The involvement of various transport channels in obesity has also been previously reported (Vasconcelos et al. 2016; Duong et al. 2018; McCauley et al. 2020). For example, a combined effect of sodium, potassium and calcium channels remodelling on atrial fibrosis was demonstrated in a diet-induced obese mouse model (McCauley et al. 2020). In the same study, mitochondrial antioxidant therapy abrogated the ion channel and structural remodelling and alleviated atrial fibrosis (McCauley et al. 2020). Interestingly, in the present study, the genes with PAS-SNPs of the Lean line were also enriched in the respiratory electron transport chain and the response to ROS.

Since obesity can be accompanied by various diseases, we next investigated whether the human orthologous genes with PAS-SNPs identified in the two lines had been previously associated with human diseases. 30 and 33 genes were identified in the Fat and Lean lines, respectively, representing more than 10% of the genes with PAS-SNPs. In both lines, a large percentage of these genes are linked to the nervous system and physical disorders, and a cluster of genes participating in immune, endocrine and metabolic diseases were also identified. The interplay between the nervous, endocrine and immune systems in obesity has already been reviewed previously (Schwartz et al. 2017; Guarino et al. 2017), as has the relationship between musculoskeletal diseases (several genes with PAS-SNPs identified in the Fat line), metabolic syndrome and obesity (Collins et al. 2018).

For all three core genes (*Nmnat1*, *Ctnna2*, *Col4a1*) in the Fat line disease network, we found that they were more highly expressed in WAT compared to the Lean line, and rs32689441 within *Nmnat1* and especially rs225665444 within *Col4a1* were identified as priority candidate PAS-SNPs. NMNAT1 (nicotinamide nucleotide adenylyltransferase 1) synthesizes NAD(+), which is required by various enzymes. NAD(+) has been demonstrated to promote preadipocyte differentiation (Okabe et al. 2020), which might be via SIRT1 (sirtuin 1) that coordinates adipogenesis (Majeed et al. 2021). Increased adipogenesis in the Fat line may also be further linked to the higher expression level of *Ctnna2* [catenin (cadherin associated protein), alpha 2], which is consistent with Greene et al. (2021), who found that *CTNNA2* expression was up-regulated in obese individuals (Greene et al. 2021). Alpha-catenin promotes adipogenesis by suppressing Wnt signalling (Laudes 2011; Sun et al. 2014). Moreover, the enlargement of obese adipocytes due to increased Fat storage is accompanied by changes in the cytoskeleton and extracellular matrix as well as in tissue structure (Pérez-Pérez et al. 2012). At the same time, the rapidly expanding adipose tissue becomes hypoxic because the vascular system cannot develop at the same rate (Halberg et al. 2009). The extracellular matrix protein COL4A1 (collagen, type IV, alpha 1) regulates angiogenesis (Maragoudakis et al. 1993) and was found to be up-regulated in vascular endothelial cells and subcutaneous white adipose tissue by hypoxia and the dominant-active form of the human hypoxia-inducible factor HIF1A (hypoxia-inducible factor 1 subunit alpha) (Manalo et al. 2005; Halberg et al. 2009). HIF1A is one of the master regulators of the cellular response to hypoxia (Kunej 2021), which has been proposed as one of the key reasons for adipose tissue dysfunction in obese individuals and the resulting inflammation and metabolic disorders (Trayhurn 2013). While the expression levels of *Hif1a* and *Hif2a* were significantly higher in the subcutaneous adipose tissue of the Fat line compared to those measured in the Lean line (Manalo et al. 2005; Morton et al. 2011), several potential regulatory variants in *Hif3a* have recently been identified in our mouse models (Mikec et al. 2022). Regarding angiogenesis, both lines carry PAS-SNPs in *Yars*, which encodes tyrosyl-tRNA synthetase that could act as an angiogenic factor depending on the splicing variant (Wakasugi et al. 2002). More importantly, a prioritised PAS-SNP rs260246262 has been identified within *Itga7* (integrin alpha 7), which expression is higher in WAT of the Fat line. ITGA7 transmits signals from extracellular matrix deposition and activates phosphorylation of intracellular FAK—JNK/ERK1/2 signals, promoting adipogenesis in WAT (Chen et al. 2022).

Meanwhile, in the Lean line, the core genes of the disease network are *Nf2* (neurofibromin 2), *Lrp1* (low-density lipoprotein receptor-related protein 1) and *Ifngr2* (interferon gamma receptor 2), which we found here to be differentially expressed (higher in the WAT of Fat line), but the PAS-SNPs do not seem to have any influence here on the APA. Nevertheless, IFNGR2 is part of the interferon-γ receptor complex involved in the JAK (Janus kinase)/STAT (signal transducer and activator of transcription) signalling pathway, which may contribute to adipocyte dysfunction and insulin resistance (Gurzov et al. 2016), the latter possibly occurring in our Fat line (Pirman et al. 2021). In addition, the expression level of *Lat* (linker for activation of T cells), which carries a priority candidate PAS-SNP rs33079159 in the Lean line, was lower in the Fat line WAT. Depletion of T cells has recently been observed in the adipose tissue of obese individuals (Porsche et al. 2021). It is possible that differential expression of *Ifngr2* and *Lat* contributes to the chronic, low-grade inflammation of adipose tissue that has been reported previously and links obesity to type II diabetes (Zatterale et al. 2020). Consistent with this, other disease-related genes that were found differentially expressed in WAT, although their regulation does not appear to be under the control of PAS-SNPs, were also found: *Kif1b* (kinesin family member 1B) and *Cdkal1* (CDK5 regulatory subunit associated protein 1-like 1), both of which have been previously associated with insulin sensitivity, diabetes and obesity in humans (Steinthorsdottir et al. 2007; Palsgaard et al. 2009; Kang et al. 2020; Maruyama et al. 2022).

Also worth mentioning are four other disease-related genes carrying PAS-SNPs with differential expression in adipose tissue: *Fgf14* (fibroblast growth factor 14), *Slc9a9* [solute carrier family 9 (sodium/hydrogen exchanger), member 9], *Tcf4* (transcription factor 4) and *Trak1* (trafficking protein, kinesin binding 1), although PAS-SNPs may not be involved in regulating their expression here. Importantly, all of these genes have been linked to neurological disorders (van Spronsen et al. 2013; Forrest et al. 2014; Di Re et al. 2017; Patak et al. 2020). In addition, *Tcf4* is a member of the microphthalmia-associated transcription factor family involved in nutrient sensing and energy homeostasis (Martina et al. 2014). According to Blaszkiewicz et al. (2019), the differential expression levels of these genes may indicate alterations in the communication between brain and adipose tissue and information transfer about the adipose tissue energy status of the Fat line.

Both lines carry PAS-SNPs in *Trim11* (tripartite motif-containing 11). TRIM11 negatively regulates interferon-β which plays a crucial role in innate immunity (Lee et al. 2013). *Trim11* was not found to be differentially expressed in our study, but the tissues where this gene is mainly expressed (embryonic stages, immune cells, testis) were not analysed here. The PAS-SNPs in *Trim11* of the two lines may therefore differentially affect the transcript translational potential in these other tissues not examined yet. PAS-SNP rs36827001 in the 3′ UTR of *Myd88* (myeloid differentiation primary response gene 88) of the Lean line was also identified. MYD88 signalling is critical for the control of both innate and adaptive immune responses to various central nervous system infections (Butchi et al. 2015). Recent studies have shown that MYD88 participates in and influences the COVID-19 disease severity (Cuevas et al. 2021; Mabrey et al. 2021).

There is a considerable interplay between hormones from the adrenal glands and adipose tissue (Kargi and Iacobellis 2014). Dysregulation of adrenal cortex contributes to insulin resistance and obesity onset (Roberge et al. 2007). In the present study, PAS-SNPs were identified in *Creb5* (cAMP responsive element-binding protein 5) and *Ppargc1a* (peroxisome proliferator-activated receptor gamma coactivator 1-alpha) in both lines and in the Fat line, respectively. We found that the expression levels of both transcripts were lower in the adrenals of the Fat line, but this was probably not due to the PAS-SNPs. It was found that cAMP-CREB-PGC1a signalling is involved in mitochondrial biogenesis (Xing et al. 2017), suggesting a reduced number of mitochondria in the Fat line adrenal glands. PAS-SNPs were also identified in *Mnat1, Mief1* and *Mrpl3* of the Fat, Lean and both lines (no difference in expression). MNAT1 (menage a trois 1) physically associates with PGC1a and is required for its transcriptional function (Sano et al. 2007). Meanwhile, *Mief1* (mitochondrial elongation factor 1), which lies in the dominant Fob2 (Fat line obesity QTL 2) confidence interval identified in our previous study (Horvat et al. 2000), and *Mrpl3* (mitochondrial ribosomal protein L3) encode proteins involved in mitochondrial translation and biogenesis. The reduced number of mitochondria has been observed, for example, in steroidogenic tissue (Leydig cells) from mice lacking insulin and IGF1 (insulin-like growth factor 1) receptors (Radovic et al. 2019).

We also found three other genes with PAS-SNPs that are differentially expressed in the adrenal glands: *Pcsk5* (Lean line), *Tenm4* (both lines) and *Lhfpl3* (Fat line), with higher, lower and higher expressions in the Fat line, again likely without the functional effect coming from their PAS-SNPs. While PCKS5 has been associated with cholesterol metabolism (Iatan et al. 2009), TENM4 (teneurin transmembrane protein 4) has been implicated in neurite development via activation of the focal adhesion kinase (FAK) signalling pathway (Suzuki et al. 2014). According to Chuang et al. (2019), decreased FAK activation can lead to cellular senescence of the adrenal gland in the Fat line. In addition, both teneurins and LHFPL3 (lipoma HMGIC fusion partner-like 3) have been shown to regulate GABA_A_ receptors (γ-Aminobutyric acid type A) (Yamasaki et al. 2017; Li et al. 2020), which are involved in catecholamine secretion in chromaffin cells of the adrenal medulla (Harada et al. 2016). Impaired catecholamine-mediated signalling has already been linked to obesity (Zouhal et al. 2013; Kargi and Iacobellis 2014). In short, these findings suggest adrenal dysfunction in the Fat line.

Furthermore, PAS-SNPs within *Arhgef4* [Rho guanine nucleotide exchange factor (GEF) 4] and *Smyd3* (SET and MYND domain containing 3) identified in both lines might have a differential effect on pre-mRNA processing and/or protein function as they are located in PAS hexamers of different APA sites within a given gene. In addition, PAS-SNPs have also been identified in other obesity-related genes: *Abcc6* and *Npc1* in the Fat line and *Lsamp* (limbic system-associated membrane protein) in the Lean line, as well as in *Arhgap8* (Rho GTPase activating protein 8) in the Fat line, which is localised in the dominant Fob2 (Horvat et al. 2000). However, their expression was not different between the two lines. Nevertheless, ARHGEF4, ARHGAP8 and LSAMP are involved in nervous system development and have been associated with neurobehavioral disorders (Koido et al. 2012; Gimelli et al. 2014; Cuellar Barboza et al. 2017). *Abcc6* (ATP-binding cassette, sub-family C (CFTR/MRP), member 6) and *Npc1* (NPC intracellular cholesterol transporter 1) are genes that encode membrane proteins involved in cholesterol transport and homeostasis (Fletcher et al. 2014; Kuzaj et al. 2014). For example, mutations in *NPC1* have been linked to early-onset and morbid obesity in adulthood (Fletcher et al. 2014). The discrepancy between our mouse models in the expression of genes participating in cholesterol metabolism and transport has been noted previously (Stylianou et al. 2005; Simončič et al. 2008, 2011). In addition, studies indicate that 65% of the variation in obesity is genetic. However, genes associated with obesity risk are easy targets for epigenetic mutations (Lavebratt et al. 2012). SMYD3 (SET and MYND domain containing 3), which methylates various histone and non-histone targets (Bottino et al. 2020), is an obesity-related gene and has been proposed as one of the candidates for body mass index in pigs and humans (Zhou et al. 2016), suggesting that PAS-SNPs in *Smyd3* may influence differential epigenetic regulation between the two lines.

Finally, we investigated whether PAS-SNPs are also found in the genes involved in APA. In addition to *Mnat1* mentioned above, seven other genes (Fat line: 2, Lean line:5) with PAS-SNPs were identified: *Rbms2* (RNA-binding motif, single-stranded interacting protein 2), *Polr2c* [polymerase (RNA) II (DNA directed) polypeptide C] of the Fat line and *Snd1* (staphylococcal nuclease and tudor domain containing 1), *Eny2* (ENY2 transcription and export complex 2 subunit), *Ints11* (integrator complex subunit 11), *Dhx15* [DEAH (Asp-Glu-Ala-His) box polypeptide 15] and *Eif3e* (eukaryotic translation initiation factor 3, subunit E) in the Lean line. However, we only found that *Eny2* was differentially expressed (higher in the mWAT of the Fat line), but with an unlikely effect of PAS-SNP. ENY2 (ENY2 transcription and export complex 2 subunit) is a multifunctional transcription factor that links transcription to mRNA export (Gurskiy et al. 2010) and participates in processing the 3′ end of transcripts (Kopytova et al. 2010). Recently, ENY2 was demonstrated to regulate the activities of multiple deubiquitinating enzymes (Atanassov et al. 2016). Interestingly, defective regulation of the ubiquitin/proteasome system in the hypothalamus of obese male mice has been proposed as an important mechanism for the progression and self-perpetuation of obesity (Ignacio-Souza et al. 2014). It is also worth highlighting two more genes with PAS-SNPs, notwithstanding the fact that their expression is not differential between the lines: *Ints11* (integrator complex subunit 6) and *Eif3e* (eukaryotic translation initiation factor 3, subunit E). INTS11 (along with INSTS6) is essential for adipocyte differentiation (Otani et al. 2013), and EIF3 has been demonstrated to associate with INTS6, which is involved in the degradation of certain mRNAs. More importantly, INTS11, a paralogue of CPSF73, interacts with INTS9, which is critical for integrator complex-mediated snRNA processing at the 3′ end (Albrecht and Wagner 2012; Wu et al. 2017), suggesting, according to the findings by Otani et al. (2013), differential snRNAs-mediated cellular regulation between the lines, which may partially explain their discrepancy in body fat percentage.

Taken together, PAS-SNPs within a substantial number of genes identified in our Fat and Lean mouse models may affect various psychological aspects of obesity, including angiogenesis, adipogenesis and adrenal gland dysfunction. Based on the PAS-SNPs locations within the corresponding genes, differences in their expression between the lines and biological functions, we propose PAS-SNPs rs225665444 (*Nmant1*), rs32689441 (*Col41a*) and rs260246262 (*Itga7*) in the Fat line and rs3022975 (*Car8*) and rs33079159 (*Lat*) in the Lean line as potential candidate functional PAS-SNPs that can affect the length of 3′ UTR (*Car8*, *Nmnat1*) and the lengths and thus the functions of the encoded proteins (*Col4a1*, *Itga7* and *Lat*). In detail, the rs260246262 within *Itga7* of the Fat line disrupts one of two motifs in the region, potentially allowing the entire ITGA7 protein to be encoded. A similar thing could happen with *Lat* and *Col4a1* of the Lean and Fat lines due to the presence of rs33079159 and rs32689441, respectively. Meanwhile, the 3′ UTR variant rs225665444 in the Fat line could lead to distinct 3′ UTR lengths of the *Nmnat1* transcript between the lines, and thus different abundance of RBPs- and miRNAs-target motifs. The rs225665444 causes the loss of ATTAAA (second most important PAS) of the most distal and most used APA site within *Nmnat1*. Although two overlapping canonical AATAAA motifs remain in the region, the loss of ATTAAA may result in an overall decreased attractiveness of the region to the polyadenylation machinery. Looking at the expressions of Affymetrix probes within the 3′ UTR of *Nmnat1*, the overall gene abundance is higher in the Fat line, but the lost ATTAAA motif by rs225665444 may favour a shorter transcript isoform. In contrast, rs3022975 destroys PAS within *Car8* 3′ UTR of the Lean line, which may lead to increased expression of the shorter transcript in the Fat line.

Although our study identified PAS-SNPs in numerous genes and proposed several candidate functional PAS-SNPs within genes that likely contribute to the divergent phenotypes between the lines, we here list some limitations of the study. The lists of APA- and obesity-related genes are not complete. There are probably more studies on PAS-SNPs, but the outdated MeSH terminology potentially may not allowed us to find all the studies included in the PubMed database. We focussed on SNPs within PAS sites of the reference mouse genome. However, SNPs in the Fat and Lean lines may introduce de-novo PAS within the same and other genes. We analysed only the location of PAS-SNPs along with Affymetrix probes for the selected candidate genes to prioritise PAS-SNPs.

Based on the results of this study, some future directions could include

1. Systematic whole-genome analyses of SNPs along with Affymetrix probes could be performed to identify other candidate PAS-SNPs.
2. The possible effect of PAS-SNPs on protein lengths could be analysed by Western blot.
3. Changes in transcript lengths or abundances of transcript isoforms could be analysed by RT-qPCR.
4. The results should be combined with RNA sequencing data (e. g. whole transcriptome termini site sequencing) and proteomics to identify functional PAS-SNPs with the potential phenotypic impact.

### Conclusion

In the present study, we identified 583 genes (Fat: 257, Lean: 318, both lines: 8) with PAS-SNPs in mouse selection lines for body fat percentage. A considerable portion of these genes is involved in various diseases, including obesity. In addition, PAS-SNPs were also identified in genes broadly involved in polyadenylation and 3′-end processing. The developed PAS-SNPs catalogue presents a key resource for designing future functional studies to uncover their role in APA, disease susceptibility and fat deposition in mouse.

## Supplementary Information

Below is the link to the electronic supplementary material.

Supplementary file1 (JPG 1203 kb)—Gene-orientation-dependent genomic location of alternative polyadenylation (APA) site relative to polyadenylation signal (PAS). (**A**) schematic diagram of gene selection influenced by PAS-SNP, (**B**) example of gene selection influenced by PAS-SNP. Legend: A[A/U]UAA – the most typical PAS signal, NN – dinucleotide (the most common is CA) after which CPSF73 (Cpsf3 in mouse) cut the pre-mRNA. APA cluster – variable APA site

Supplementary file2 (JPG 1386 kb)—PAS motifs in mouse genome as obtained from PolyASite 2.0, their counts, and relative abundances. (**A**) Whole-genome PAS, (**B**) PAS within genes

Supplementary file3 (JPG 314 kb)—The number of genes having PAS-SNPs identified in Fat and Lean lines

Supplementary file4 (JPG 1346 kb)—GO enrichment analysis of genes with PAS-SNPs. (**A**) Total genes (583), (**B**) genes in the Fat line (265), and (**C**) genes in the Lean line (326) (Source: MonaGO)

Supplementary file5 (JPG 880 kb)—PAS-SNPs in *Tenm4 *of Fat line (the APA site and sequence indicated red) and Lean lines (the APA site and sequence indicated green). (**A**) Location of APA and PAS sites as of PolyASite database. (**B**) Visualization of PAS-SNPs using GenomeBrowse (Golden Helix)

Supplementary file6 (JPG 930 kb)—PAS-SNPs within *Mrpl3* 3′ UTR of Fat (the APA site and sequence indicated red) and Lean lines (the APA site and sequence indicated green). (**A**) Location of APA and PAS sites as of PolyASite database. (**B**) Visualization of PAS-SNPs using GenomeBrowse (Golden Helix)

Supplementary file7 (JPG 824 kb)—**S7** Intronic PAS-SNP rs38383450 in *Ppargc1a* 3′ UTR of Lean line. (**A**) Location of APA and PAS sites as of PolyASite database. (**B**) Visualization of PAS-SNPs using GenomeBrowse (Golden Helix)

Supplementary file8 (JPG 753 kb)—PAS-SNP rs38383450 in *Eny2* 3′ UTR of Lean line. (**A**) Location of APA and PAS sites as of PolyASite database. (**B**) Visualization of PAS-SNPs using GenomeBrowse (Golden Helix)

Supplementary file9 (JPG 1380 kb)—Visual identification of potentially functional PAS-SNPs. (**A**) *Car8*, (**B**) *Col4a1*, (**C**) *Itga7*, and (**D**) *Lat*. 3^rd^ track, black rectangles – Affymetrix probes with no expression difference between the lines, red and green rectangles – the expression being higher and lower in the Fat line compared to the Lean line, respectively; red and green vertical lines denote PAS-SNPs identified in the Fat and Lean line

Supplementary file10 (XLSX 18 kb)—Search terms used for screening PubMed database for the literature related to polyadenylation, PAS-SNPs and their relationship with diseases and obesity

Supplementary file11 (XLSX 36 kb)—A set of 431 obesity-related genes obtained from the literature

Supplementary file12 (XLSX 26 kb)—A set of 281 alternative polyadenylation (APA)-related genes obtained from various databases

Supplementary file13 (XLSX 79 kb)—Differentially expressed common (genes containing PAS-SNPs in both lines), disease-associated, and obesity- and APA-related genes carrying PAS-SNPs

Supplementary file14 (XLSX 473 kb)—PAS-SNPs identified in Fat and Lean mouse selection lines
