# Supplementary figures and images for: Genome-wide screening for genetic variants in polyadenylation signal (PAS) sites in mouse selection lines for fatness and leanness
Source: Mamm Genome. 2022 Nov 21;34(1):12–31. doi: 10.1007/s00335-022-09967-8 (PMC9684942; doi:10.1007/s00335-022-09967-8)

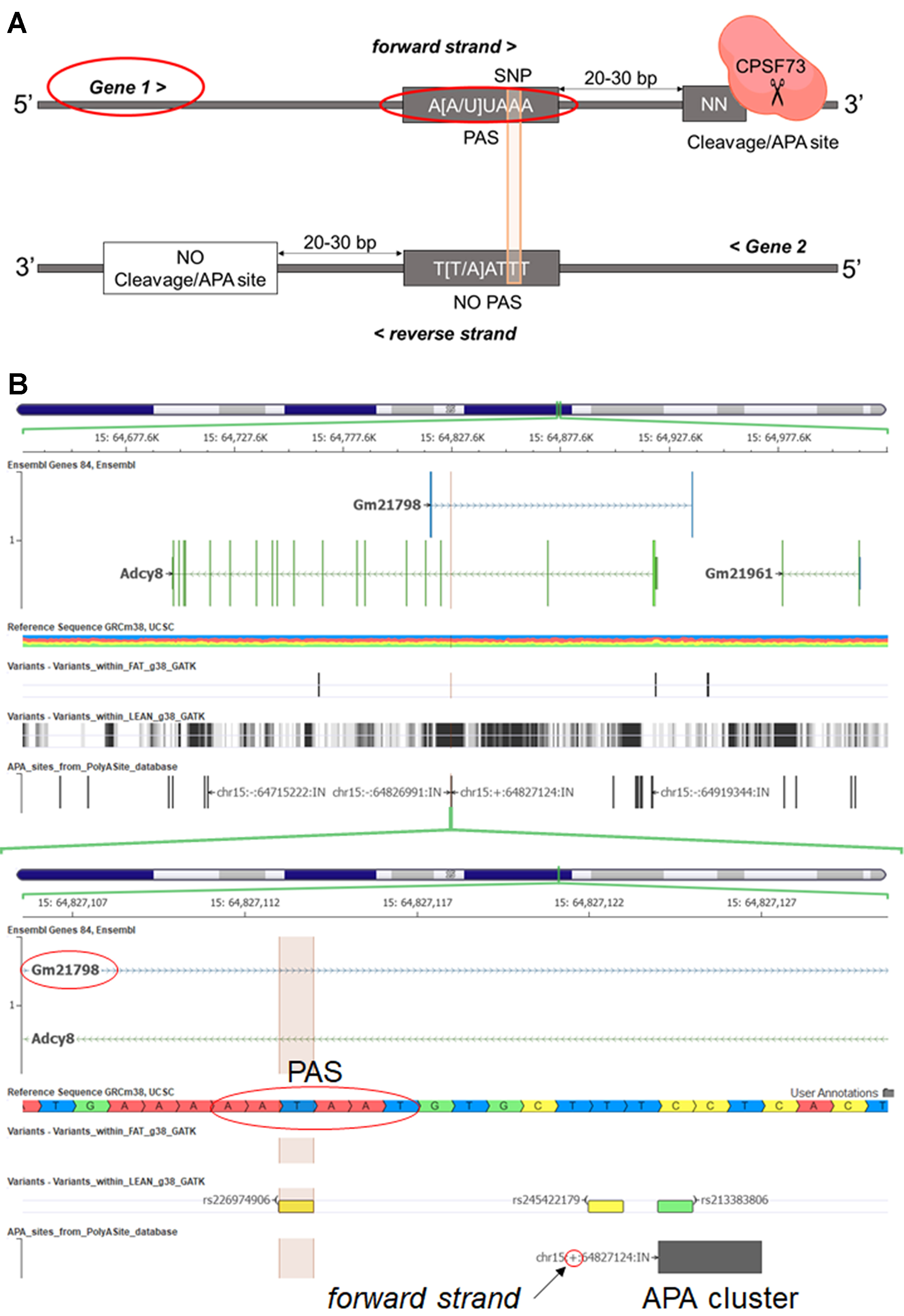

Supplement: Supplementary file 1 — Supplementary file1 (JPG 1203 kb)—Gene-orientation-dependent genomic location of alternative polyadenylation (APA) site relative to polyadenylation signal (PAS). (A) schematic diagram of gene selection influenced by PAS-SNP, (B) example of gene selection influenced by PAS-SNP. Legend: A[A/U]UAA – the most typical PAS signal, NN – dinucleotide (the most common is CA) after which CPSF73 (Cpsf3 in mouse) cut the pre-mRNA. APA cluster – variable APA site [file 335_2022_9967_MOESM1_ESM.jpg]

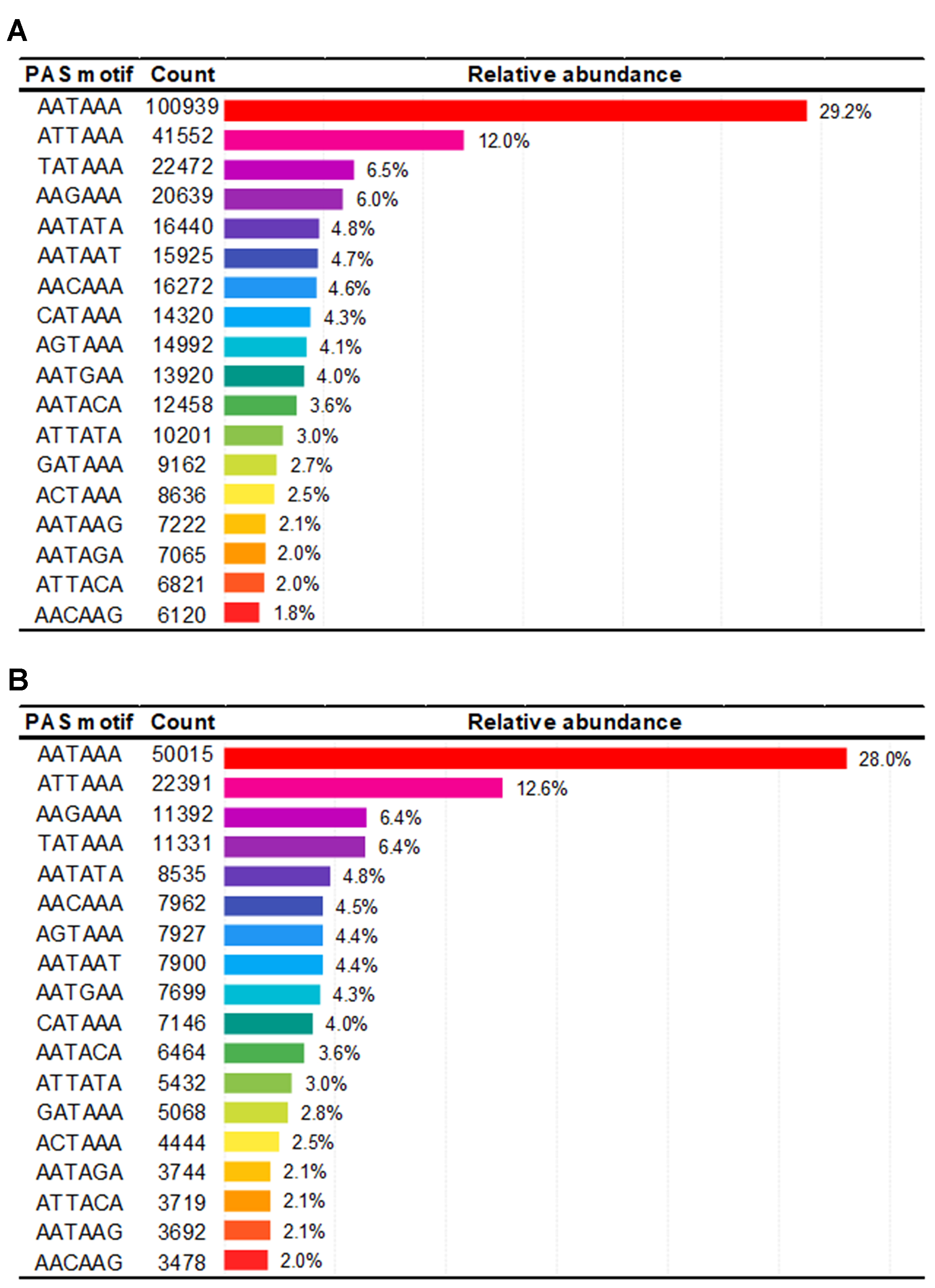

Supplement: Supplementary file 2 — Supplementary file2 (JPG 1386 kb)—PAS motifs in mouse genome as obtained from PolyASite 2.0, their counts, and relative abundances. (A) Whole-genome PAS, (B) PAS within genes [file 335_2022_9967_MOESM2_ESM.jpg]

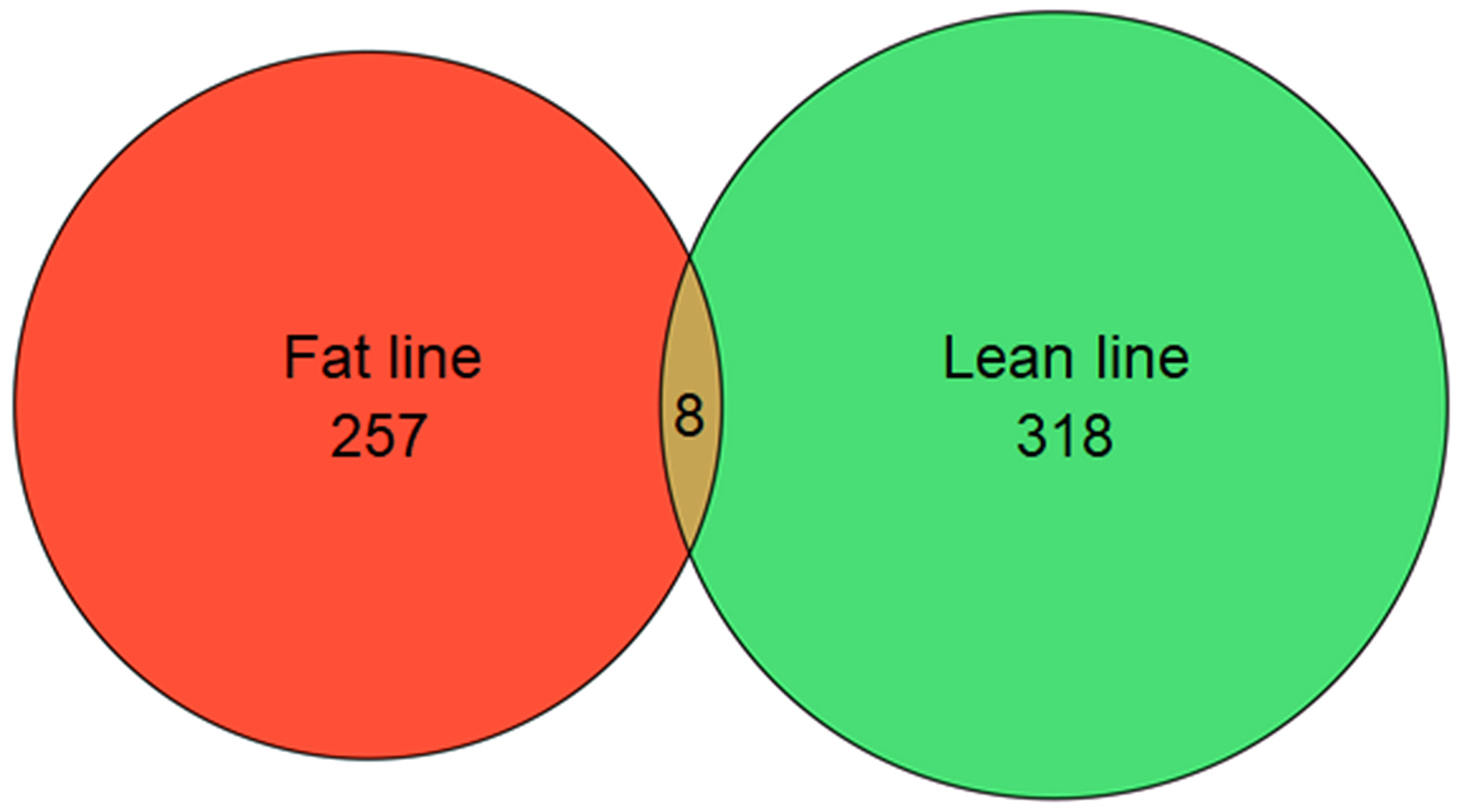

Supplement: Supplementary file 3 — Supplementary file3 (JPG 314 kb)—The number of genes having PAS-SNPs identified in Fat and Lean lines [file 335_2022_9967_MOESM3_ESM.jpg]

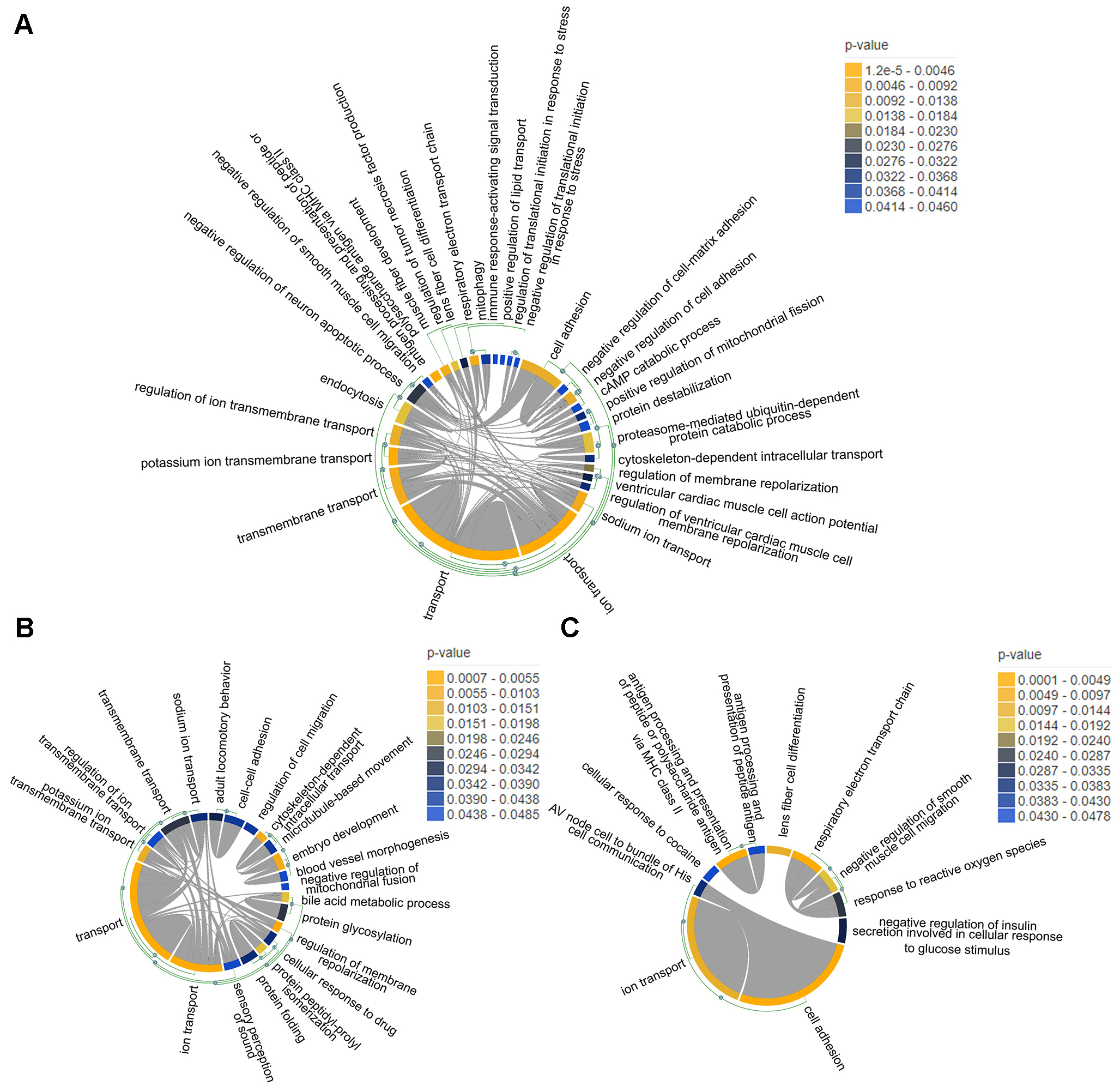

Supplement: Supplementary file 4 — Supplementary file4 (JPG 1346 kb)—GO enrichment analysis of genes with PAS-SNPs. (A) Total genes (583), (B) genes in the Fat line (265), and (C) genes in the Lean line (326) (Source: MonaGO) [file 335_2022_9967_MOESM4_ESM.jpg]

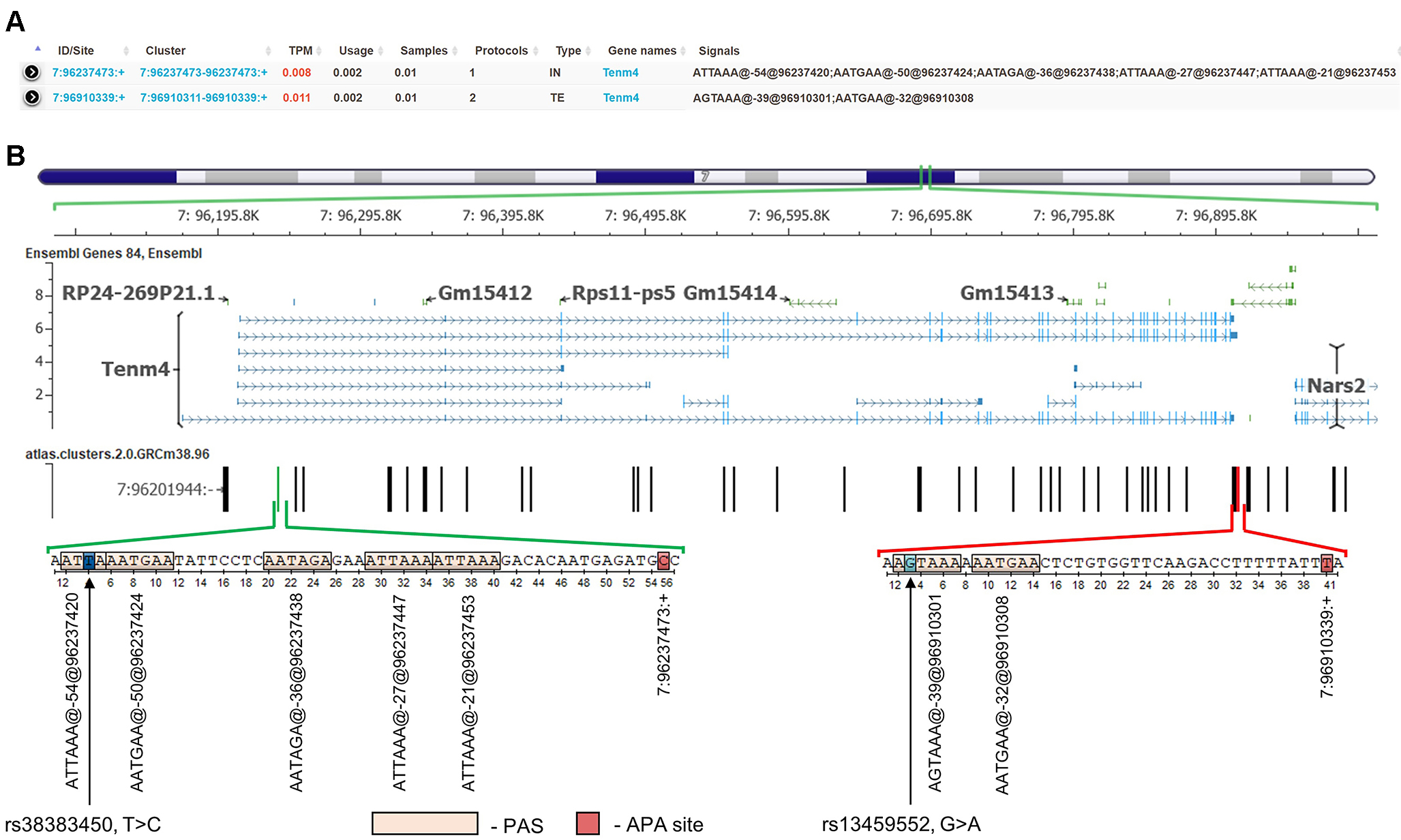

Supplement: Supplementary file 5 — Supplementary file5 (JPG 880 kb)—PAS-SNPs in Tenm4 of Fat line (the APA site and sequence indicated red) and Lean lines (the APA site and sequence indicated green). (A) Location of APA and PAS sites as of PolyASite database. (B) Visualization of PAS-SNPs using GenomeBrowse (Golden Helix) [file 335_2022_9967_MOESM5_ESM.jpg]

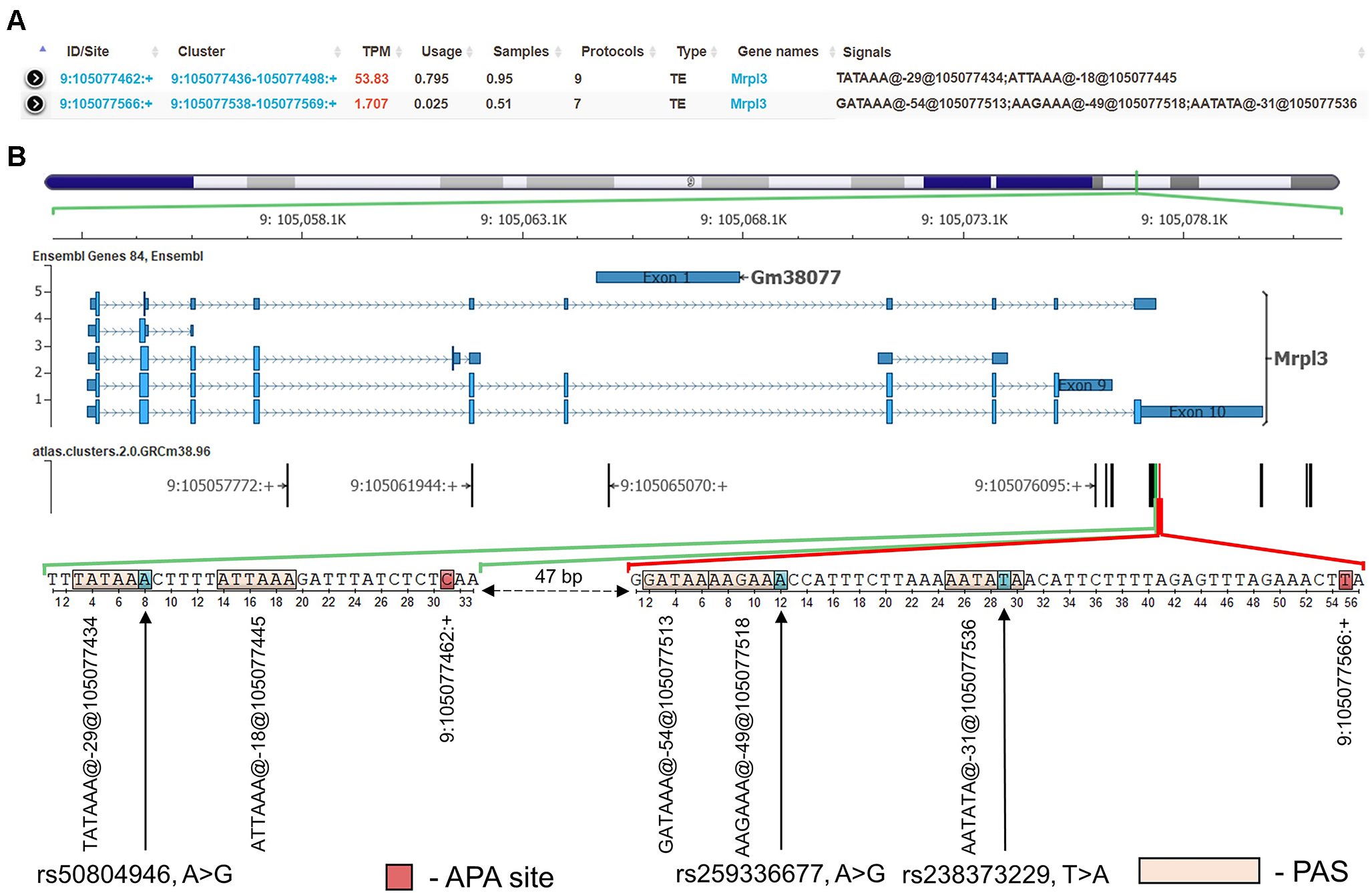

Supplement: Supplementary file 6 — Supplementary file6 (JPG 930 kb)—PAS-SNPs within Mrpl3 3′ UTR of Fat (the APA site and sequence indicated red) and Lean lines (the APA site and sequence indicated green). (A) Location of APA and PAS sites as of PolyASite database. (B) Visualization of PAS-SNPs using GenomeBrowse (Golden Helix) [file 335_2022_9967_MOESM6_ESM.jpg]

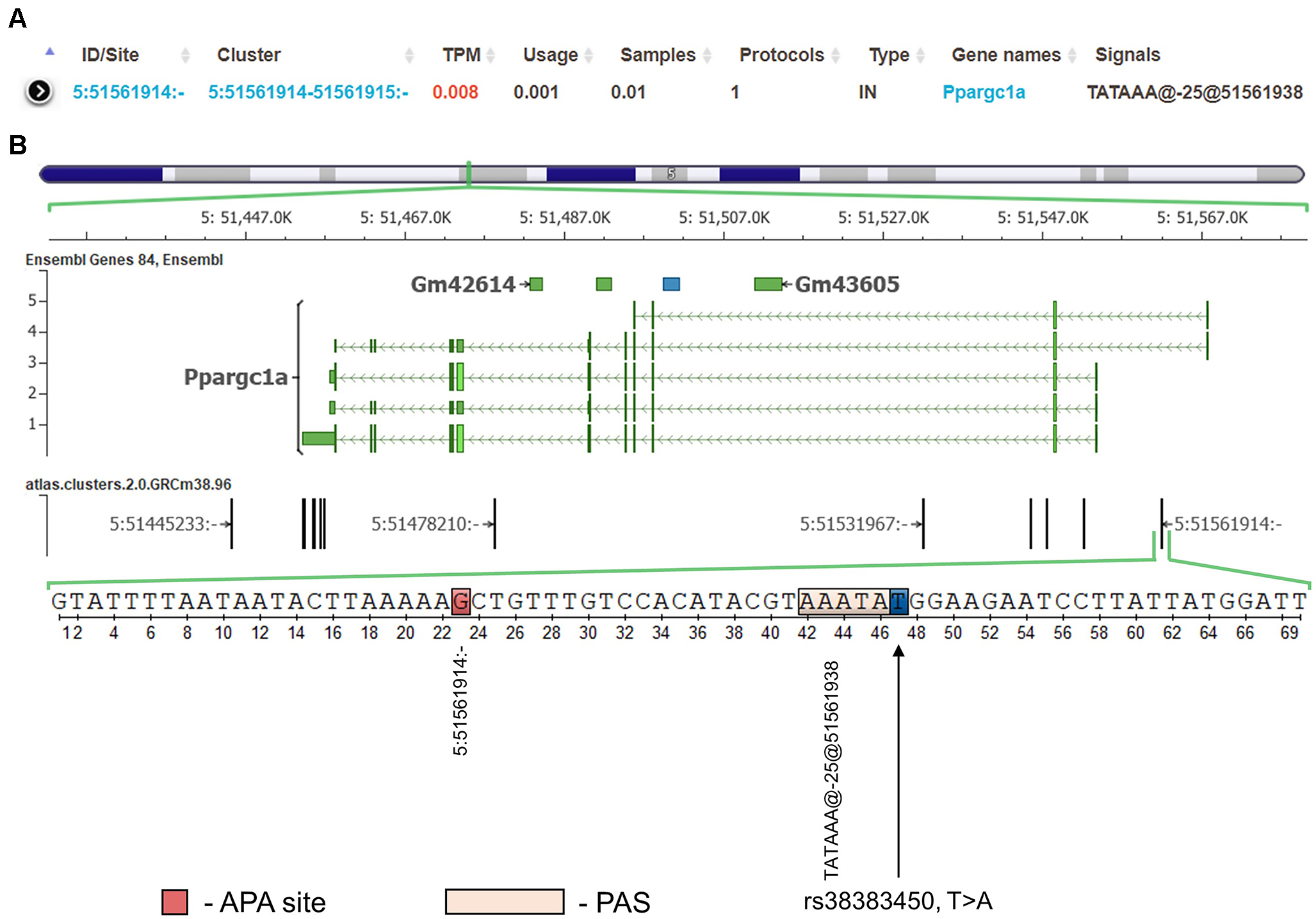

Supplement: Supplementary file 7 — Supplementary file7 (JPG 824 kb)—S7 Intronic PAS-SNP rs38383450 in Ppargc1a 3′ UTR of Lean line. (A) Location of APA and PAS sites as of PolyASite database. (B) Visualization of PAS-SNPs using GenomeBrowse (Golden Helix) [file 335_2022_9967_MOESM7_ESM.jpg]

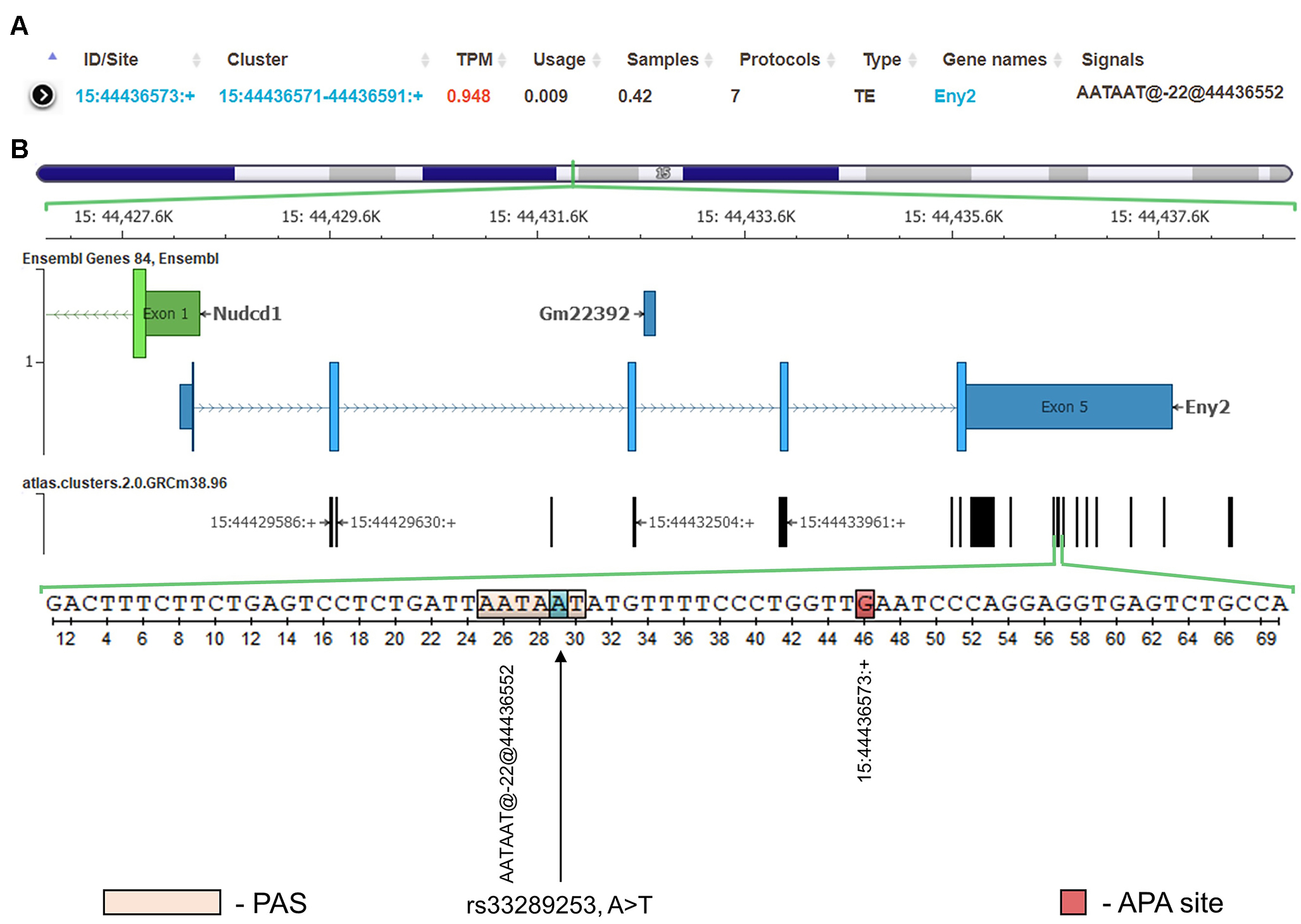

Supplement: Supplementary file 8 — Supplementary file8 (JPG 753 kb)—PAS-SNP rs38383450 in Eny2 3′ UTR of Lean line. (A) Location of APA and PAS sites as of PolyASite database. (B) Visualization of PAS-SNPs using GenomeBrowse (Golden Helix) [file 335_2022_9967_MOESM8_ESM.jpg]

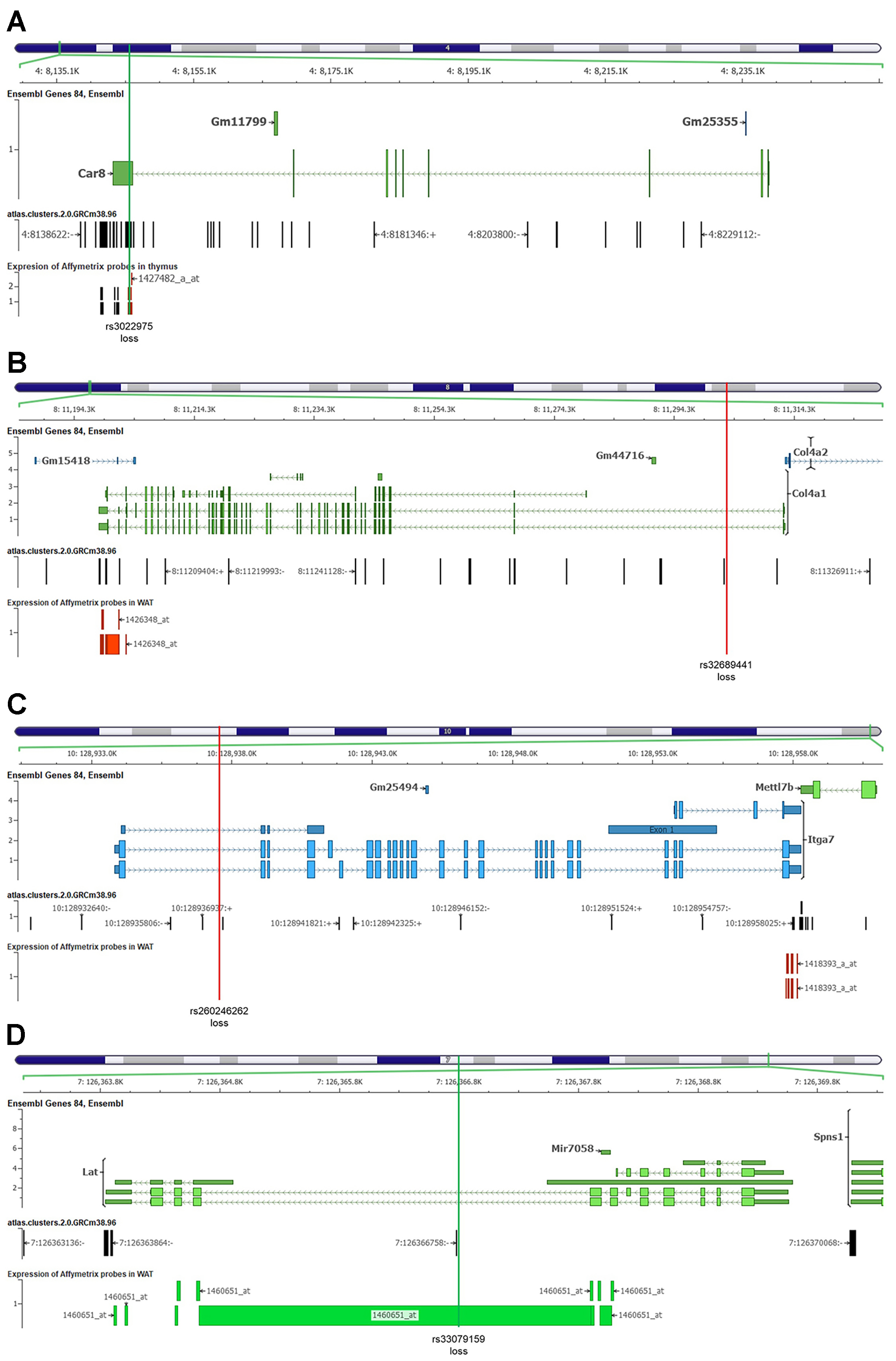

Supplement: Supplementary file 9 — Supplementary file9 (JPG 1380 kb)—Visual identification of potentially functional PAS-SNPs. (A) Car8, (B) Col4a1, (C) Itga7, and (D) Lat. 3rd track, black rectangles – Affymetrix probes with no expression difference between the lines, red and green rectangles – the expression being higher and lower in the Fat line compared to the Lean line, respectively; red and green vertical lines denote PAS-SNPs identified in the Fat and Lean line [file 335_2022_9967_MOESM9_ESM.jpg]
